# Through the looking glass: Deep interpretable dynamic directed connectivity in resting fMRI

**DOI:** 10.1016/j.neuroimage.2022.119737

**Published:** 2022-11-07

**Authors:** Usman Mahmood, Zening Fu, Satrajit Ghosh, Vince Calhoun, Sergey Plis

**Affiliations:** a Tri-institutional Center for Translational Research in Neuroimaging and Data Science (TReNDS), Georgia State University, Georgia Institute of Technology, Emory University, Atlanta, GA, USA; b Georgia State University, Department of Computer Science, Atlanta, GA, USA; c McGovern Institute for Brain Research, Massachusetts Institute of Technology, Cambridge, MA USA; d Department of Otolaryngology - Head and Neck Surgery, Harvard Medical School, Boston, MA USA; e Georgia Institute of Technology, Department of Electrical and Computer Engineering, Atlanta, GA, USA

**Keywords:** Dynamic directed connectivity, Interpretable deep learning, Resting state fMRI, Brain disorders

## Abstract

Brain network interactions are commonly assessed via functional (network) connectivity, captured as an undirected matrix of Pearson correlation coefficients. Functional connectivity can represent static and dynamic relations, but often these are modeled using a fixed choice for the data window Alternatively, deep learning models may flexibly learn various representations from the same data based on the model architecture and the training task. However, the representations produced by deep learning models are often difficult to interpret and require additional posthoc methods, e.g., saliency maps. In this work, we integrate the strengths of deep learning and functional connectivity methods while also mitigating their weaknesses. With interpretability in mind, we present a deep learning architecture that exposes a directed graph layer that represents what the model has learned about relevant brain connectivity. A surprising benefit of this architectural interpretability is significantly improved accuracy in discriminating controls and patients with schizophrenia, autism, and dementia, as well as age and gender prediction from functional MRI data. We also resolve the window size selection problem for dynamic directed connectivity estimation as we estimate windowing functions from the data, capturing what is needed to estimate the graph at each time-point. We demonstrate efficacy of our method in comparison with multiple existing models that focus on classification accuracy, unlike our interpretability-focused architecture. Using the same data but training different models on their own discriminative tasks we are able to estimate task-specific directed connectivity matrices for each subject. Results show that the proposed approach is also more robust to confounding factors compared to standard dynamic functional connectivity models. The dynamic patterns captured by our model are naturally interpretable since they highlight the intervals in the signal that are most important for the prediction. The proposed approach reveals that differences in connectivity among sensorimotor networks relative to default-mode networks are an important indicator of dementia and gender. Dysconnectivity between networks, specially sensorimotor and visual, is linked with schizophrenic patients, however schizophrenic patients show increased intra-network default-mode connectivity compared to healthy controls. Sensorimotor connectivity was important for both dementia and schizophrenia prediction, but schizophrenia is more related to dysconnectivity between networks whereas, dementia bio-markers were mostly intra-network connectivity.

## 1. Introduction

Functional connectivity has emerged as a promising tool for understanding the brain’s functional architecture and has been widely used (Greicius et al., 2003; Lee et al., 2013; Rogers et al., 2007; Van Den Heuvel and Pol, 2010a). Disruptions in the brain’s functional connectivity are often linked to brain disorders evident in patients’ behavior (van den Heuvel and Pol, 2010b). For example, schizophrenic patients have high level of functional dysconnectivity between brain networks (Culbreth et al., 2021; Fu et al., 2017; Lynall et al., 2010; Morgan et al., 2020; van den Heuvel et al., 2010; Yu et al., 2011; Zhang et al., 2019; Zhu et al., 2020) and exhibit dysregulated dynamic connectivity across multiple brain networks (Supekar et al., 2019). Alzheimer’s disease (AD) is also known to disrupt brain dynamics leading to wide-spread cognitive dysfunction (Haan et al., 2011).

The association of brain disorders with abnormal static or dynamic functional connectivity highlights the need to develop models that can identify disorder-specific connectivity aberrations. This observation guides development of various approaches to brain connectivity analysis (Arslan et al., 2018; Kazi et al., 2021; Kim and Ye, 2020; Ktena et al., 2017; 2018; Ma et al., 2019; Parisot et al., 2018; Yan et al., 2017). However in most existing approaches, the functional connectivity matrices are not informed by the prediction task but instead estimated prior to training; thus, they depend entirely on the chosen input window of data samples. The independence from the downstream task results in inflexible estimation of connectivity matrices as the estimate is unchanged regardless of whether the task is to predict a brain disorder, age, or other quantity. Kim et al. (2021) proposed a method where the functional connectivity structure is computed based on the learned representations of the data, but even this method lacks a learnable connectivity estimation method. We argue that task-dependent connectivity matrices can be estimated by a deep learning (DL) model using learnable weights. DL models are flexible in their ability to learn a variety of representations from the same data based on the architecture and ground-truth signal used in training.

However, using a DL method to estimate a connectivity matrix can be challenging without the presence of the ground-truth graph during training. Another problem of many DL models is lack of consistency and interpretability in the learned representations. Saliency maps commonly used to address interpretability of these models (Angelov et al., 2021; Lewis et al., 2021; Ras et al., 2021; Simonyan et al., 2014) may be difficult to interpret (Liu et al., 2021). Arguably, the difficulty of interpreting representations is the reason why studies using DL models incorporate inflexible but interpretable feature selection steps for connectivity estimation, for example Pearson correlation coefficients (PCC) (Freedman et al., 2007).

In most of the current studies, functional connectivity estimates are either static or dynamically computed using a sliding window approach dependent on the window size and stride (Armstrong et al., 2016; Damaraju et al., 2014; Fu et al., 2020; 2018; Gadgil et al., 2021; Yao et al., 2020). Unable to capture non-stationarity, static matrices miss essential information about dynamics. For example, dynamic functional connectivity estimates show re-occurring patterns which cannot be captured by their static counterparts (Allen et al., 2012; Calhoun et al., 2014; Hutchison et al., 2013). Using a static graph learning method to capture a dynamical system may reduce classification performance (Xu et al., 2020). Kipf et al. (2018) show improved results by just dynamically re-evaluating the learned static graph during testing. The improved performance for the relevant task is understandable as the dynamic connectivity provides essential information about the system, for instance, capturing re-occurring patterns. The brain’s functional activity is also perceived to be highly dynamic and hence cannot be faithfully captured with a static or even window-based approach (Yaesoubi et al., 2018).

Furthermore, studies using functional connectivity to measure connectivity between brain regions or networks do not capture the direction of interaction and only measure undirected statistical dependence such as correlations, coherence, or transfer entropy. Correlation can arise for many reasons; for example, due to a common cause when an unobserved network affects two networks that are observed (Pearl, 2000; Spirtes et al., 1993). Arguably, dynamics of interaction among brain networks is beyond simple correlations and correlation may only partially describe it. Whereas, effective connectivity is a more general way to represent dynamic and directed relationships among brain’s intrinsic networks. As introduced by Friston (2011) effective connectivity falls into a model-based class of methods while multiple other methods, including those in the model-free class have been since developed (Bielza and Larranaga, 2014; Chiang et al., 2017; Chickering, 2002a; 2002b; Deshpande et al., 2011; Goebel et al., 2003; Gorrostieta et al., 2013; Mitra et al., 2014; Schreiber, 2000; Seth et al., 2015; Spirtes and Glymour, 1991; Ursino et al., 2020; Vicente et al., 2011).

Like these approaches, to estimate brain networks’ connectivity that is 1) directed, 2) interpretable, 3) flexible, and 4) dynamic, we have developed an approach called the Directed Instantaneous Connectivity Estimator (DICE): a predictive model to estimate dynamic directed connectivity between brain networks, represented as a dynamically varying directed graph by predicting the downstream binary label. Our model may be placed into the category of model-free connectivity methods as it does not model the data generation process. We defer to using “directed (network) connectivity” (D(N)C) for the graphs that DICE estimates.

Unlike existing supervised DL models that typically produce difficult-to-interpret representations, we designed our model primarily with interpretability in mind. Our model reveals what it learned about the dynamics of brain network connectivity without using post hoc interpretability methods. Effectively, we have built a “glass-box” layer within a traditionally “black-box” DL model. In contrast to commonly used hidden layers, the “glass-box” layer propagates a weighted adjacency matrix of a directed graph, ensuring that it is interpretable in the context of the classification task. Hence, by estimating DC based on the task and using only the estimated connectivity structure for classification, our model learns to capture task-relevant networks and their connectivity, leading to a flexible estimation of an interpretable DC. By estimating DC instantaneously (window-size = 1), DICE removes the need for the window-size parameter used in many dynamic connectivity studies.

To thoroughly validate DICE’s performance, we conduct a series of experiments on four neuroimaging datasets that span three disorders (schizophrenia, autism, and dementia) and cover a wide age range. We train the model on classification tasks for each of these brain disorders, age prediction, and gender classification, and analyze the resulting DC of the “glass-box” layer. Surprisingly, our deliberate focus on stable interpretable results has an enhancing side effect on DICE’s predictive performance. As we show, the model’s predictions are better or on par with state-of-the-art methods that were developed with a focus on classification performance rather than interpretability. We show that when learning to classify subjects based on a specific criterion, DICE estimates interpretable DCs specific to that criterion. For gender and mental disorder classification, subgraphs emphasized by the learned DCs are discriminative of gender and mental disorders, respectively. We also demonstrate that DICE learns interpretable DCs distinct to dementia, gender, and age prediction for the same subjects by enhancing connectivity for networks that pertain to the training signal. Our flexible estimation of DC structures advances the results of Salehi et al. (2020), which show that functional parcel boundaries change for an individual based on the cognitive state. We show an increased utility of the inferred directionality for increasing the precision of explainable group differences. As a result, DICE can resolve more states in fMRI dynamics than is resolvable in typical dynamic functional network connectivity analyses. Additionally, DICE incorporates a temporal attention module that highlights crucial time steps relevant to the task, further improving the interpretation of predictions for the dynamics. The learned DC structures and temporal attention weights are stable and consistent across randomly-seeded trials.

## 2. Materials and methods

### 2.1. Materials

We use resting state functional magnetic resonance imaging (rsfMRI) data as input to our model. fMRI measures blood oxygenation level-dependent (BOLD) signal, which captures the functional activity of the brain over time. We test our model by classifying three different brain disorders, predict gender and age of subjects. For each brain disorder we perform binary classification of healthy controls (HC) and patients. Four datasets used in this study are collected from FBIRN (Function Biomedical Informatics Research Network^1^) Keator et al. (2016) project, from release 1.0 of ABIDE (Autism Brain Imaging Data Exchange^2^) Di Martino et al. (2014) and from release 3.0 of OASIS (Open Access Series of Imaging Studies^3^) Rubin et al. (1998). Healthy controls from the HCP (Human Connectome Project) (Van Essen et al., 2013) are used for gender prediction. Refer to Table 1 for details of the datasets.

#### 2.1.1. Preprocessing

We use two typical brain parcellation techniques; independent component analysis (ICA) and regions of interest (ROIs) based on a predefined atlas. The preprocessing pipeline used depends on the parcellation technique and the pipeline used in state-of-the-art studies for the dataset. All the preprocessing was done before training the model.

##### ICA parcellation:

For all experiments conducted using ICA as brain parcellation technique the fMRI data was preprocessed using statistical parametric mapping (SPM12, http://www.fil.ion.ucl.ac.uk/spm/) under the MATLAB 2021 environment. A rigid body motion correction was performed to correct subject head motion, followed by the slice-timing correction to account for timing difference in slice acquisition. The fMRI data were subsequently warped into the standard Montreal Neurological Institute (MNI) space using an echo planar imaging (EPI) template and were slightly resampled to 3 × 3 × 3 mm^3^ isotropic voxels. The resampled fMRI images were then smoothed using a Gaussian kernel with a full width at half maximum (FWHM) = 6 mm.

We selected subjects for further analysis (Fu et al., 2021) if the subjects have head motion ≤ 3° and ≤ 3 mm, and with functional data providing near full brain successful normalization (Fu et al., 2019). 100 ICA components are estimated using a novel fully automated Neuromark pipeline “neuromark_fmri_1.0”^4^ described in Fu et al. (2019). This method is capable of capturing robust imaging features that are comparable across subjects, datasets, and studies, which is beneficial for those studies need replication. The Neuromark framework leverages an adaptive-ICA technique that automates the estimation of comparable brain markers across subjects, datasets, and studies. A set of component templates were used as references to guide the estimation of single-scan components for the data. These component templates were created via a unified ICA pipeline. They were constructed using an independent resting-state fMRI data with large samples of healthy subjects from the genomics superstruct project (GSP). The GSP data include 1005 subjects’ scans that passed the data QC. High model order (order = 100) group ICA was performed on the GSP data, and then the independent components (ICs) from the GSP data were used as the references to extract components for each dataset used for experiment in this study. The Neuromark framework extracts the components for each subject respectively, which means that the estimation of features of each subject is not influenced by the others. However, the choice of components (and number of components) can influence accuracy, but our study is not focusing on determining the best number of ICs rather use the available components and let the model decide the task-dependant components.

##### Region parcellation:

State-of-the-art methods use different preprocessing pipelines for different datasets. For comparison with these methods on HCP, ABIDE, and FBIRN datasets, we select the same preprocessing pipelines as in the relevant comparing method. We use the HCP (Van Essen et al., 2013) data which was first minimally pre-processed following the pipeline described in Glasser et al. (2013). The preprocessing includes gradient distortion correction, motion correction, and field map preprocessing, followed by registration to T1 weighted image. The registered EPI image was then normalized to the standard MNI152 space. To reduce noise from the data, FIX-ICA based denoising was applied (Griffanti et al., 2014; Salimi-Khorshidi et al., 2014). To minimize the effects of head motion subject scans with framewise displacement (FD) over 0.3mm at any time of the scan were discarded. The FD was computed with fsl motion outliers function of the FSL (Jenkinson et al., 2012). There were 152 discarded scans from filtering out with the FD, and 942 scans were left. For all experiments, the scans from the first run of HCP subjects released under S1200 were used. ABIDE (Di Martino et al., 2014) was pre-processed using C-PAC (Aertsen and Preissl, 1991). The preprocessing includes; slice time correction, motion correction, skull striping, global mean intensity normalization, nuisance signal regression, band pass filtering, and finally functional images were registered to anatomical space (MNI12). After preprocessing using C-PAC, 871 out of 1112 subjects were chosen based on the visual quality, inspected by three human experts which looked for brain coverage, high movement peaks and other artifacts resulted by scanner (Abraham et al., 2017; Cao et al., 2021; Parisot et al., 2018). To pre-process FBIRN data, SPM12 pipeline was used as explained in previous section with few extra steps. After the smoothing using a Gaussian kernel, the functional images were temporally filtered by a finite impulse response (FIR) bandpass filter (0.01 Hz-0.15 Hz). Then for each voxel, six rigid body head motion parameters, white matter (WM) signals, and cerebrospinal fluid (CSF) signals were regressed out using linear regression.

We used two atlases for brain parcellation; Schaefer et al. (2017), and Harvard Oxford (HO) (Desikan et al., 2006) with 200, and 111 regions respectively. For each region, average value is computed for all the voxels falling inside a region, thus resulting into a single time-series for each region. After dividing data into regions, each time-series was standardized by their zscore having zero mean and unit variance.

### 2.2. Method

**Fig. 1.**
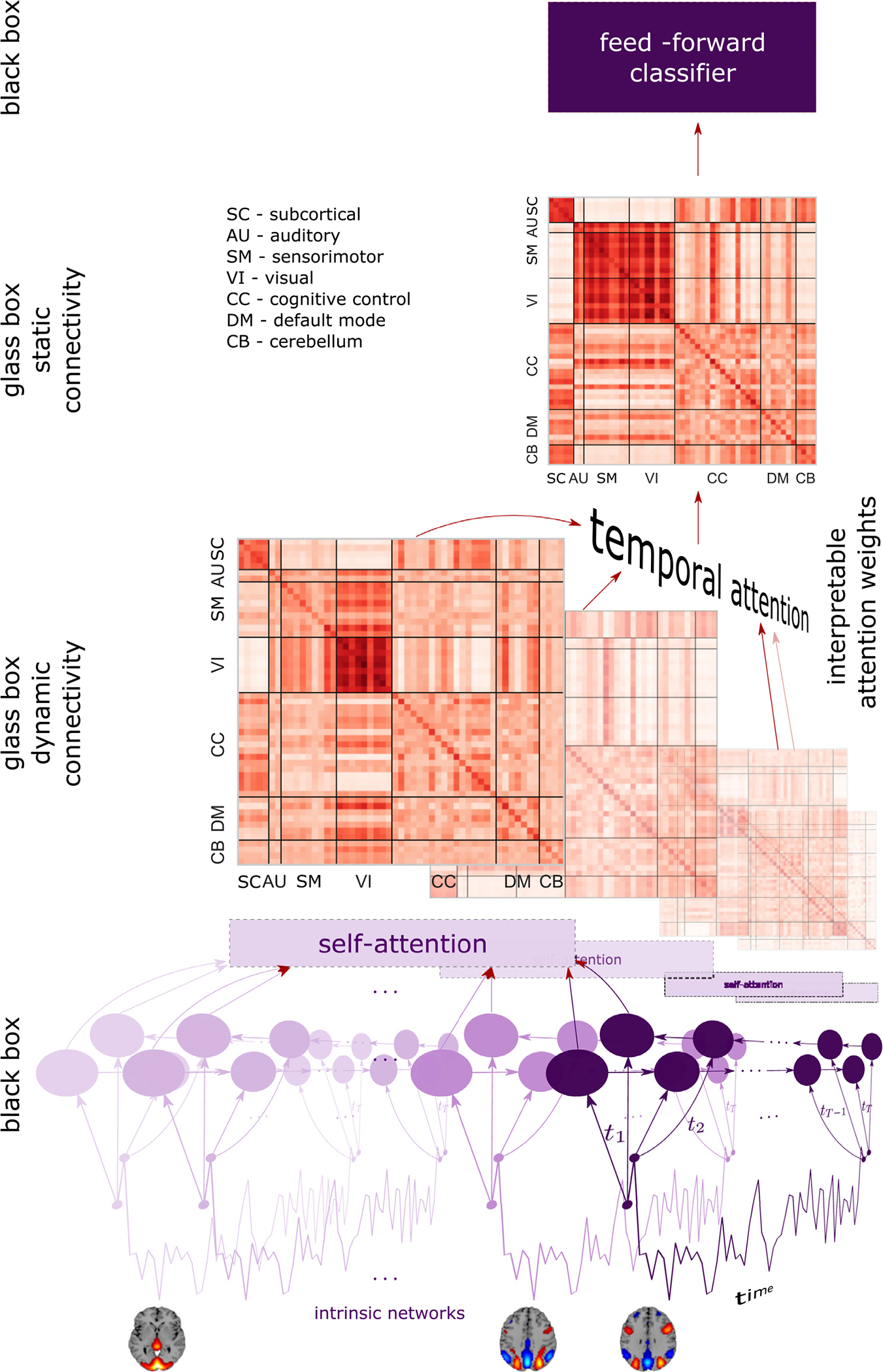
DICE architecture using biLSTM, self-attention and temporal attention. We use self-attention between the embeddings of all components/nodes at each time-point to estimate the DC **W**_*i*_. Temporal attention is used to create a weighted sum of the *T* DC. Architecture details of temporal attention is shown in Fig. 2.

Our DICE model recieves the time-courses of the ICA components or ROIs represented as a matrix of size *N* * *T* (Number of components/ROIs * Number of time-points) and learns a set of *T* directed graphs representing the dynamic DC or DNC between spatial components (e.g., ICA-based spatial components, regions from an atlas), which we designate as nodes of a graph by predicting the binary labels. Let *G* represent the set of graphs where *G* = {*g*_1_, *g*_2_, … *, g*_*T*_} where *T* is the total time-points and *g*_*t*_ = (*V*_*t*_, *E*_*t*_), where, *V*_*t*_ and *E*_*t*_ represent the nodes and edges present at time-point *t*. To create the graph *g*_*t*_ we first use a bidirectional long short-term memory (biLSTM) (Schuster and Paliwal, 1997) module to create the embedding $h_{t}^{i}$ of node *i* at time *t*. We then use a self-attention module (Vaswani et al., 2017) which takes all such embeddings at each time *t* and create a weight matrix among nodes thus providing the DC (graph) between nodes at each time-point. To create a final graph *G*^*f*^ for downstream classification, we use a temporal attention model that assign a weight to each *g*_*t*_ and compute the weighted sum of the set *G*. We explain the working and purpose of each module in detail in the following sections. Figure 1 shows the complete architecture.

#### 2.2.1. biLSTM

The time-point value $x_{t}^{i}$ for node *i* at time *t* can be effected by many different factors and relations. Capturing these relations can increase model interpretability and improve downstream classification performance. In a time-series (fMRI data), one of these factors is the values/data at previous time-points $x_{1\ldots t-1}^{i}$. In fMRI data, this relationship is unknown and is hard to capture and hence cannot be computed using a fixed method/formula (hand-crafted features). The difficulty is further increased by a) low temporal resolution of fMRI data and b) the fact that it is unknown how farther in time the effects of a time-point remains in a time-series. These effects are different for each subject and can even vary among nodes of the same subject. LSTMs have proved to be extremely effective for time-series/sequence data where the model takes an input from a sequence at time-point *t* and create representation for current and also predict representation for future time-courses based on the representation of previous time-points. LSTMs learn the temporal relationships between data through the cell’s memory and forget gate. These gates are optimized on the data and downstream task (ground-truth signal) and the relationships between data are learned instead of computed. The working of the LSTMs can be explained by the following set of equations. *σ* represents sigmoid activation, and ⊙ is the Hadamard product (Million, 2007).


(1) $$i_{t}=\sigma \left(W_{ii}x_{t}+b_{ii}+W_{hi}h_{t-1}+b_{hi}\right)\;f_{t}=\sigma \left(W_{if}x_{t}+b_{if}+W_{hf}h_{t-1}+b_{hf}\right)\;g_{t}=\tanh\left(W_{ig}x_{t}+b_{ig}+W_{hg}h_{t-1}+b_{hg}\right)\;o_{t}=\sigma \left(W_{io}x_{t}+b_{io}+W_{ho}h_{t-1}+b_{ho}\right)\;c_{t}=f_{t}⊙c_{t-1}+i_{t}⊙g_{t}\;h_{t}=o_{t}⊙\tanh\left(c_{t}\right)$$

In the above equations, **i**_*t*_, **f**_*t*_, and **o**_*t*_ represent the input, forget and output gates at time *t* respectively. **c**_*t*_ represents the cell state (memory), **g**_*t*_ represents candidate for the cell state, and **h**_*t*_ represents the representation/embedding for the input at *t*. **W**_*ix*_ and **W**_*hx*_ represent the weights for the input and hidden vectors for the respective gate *x* ∈ {*i*-input*, f*-forget*, o*-output}. Similarly *b*_*ix*_, *b*_*hx*_ are the biases for the respective gate *x* ∈ {*i, f, o*}. We use a biLSTM to create representation **h**_*t*_ for each node *i*. Thus $h_{t}^{f}=LSTM\left(x_{t},h_{t-1}\right)$, $h_{t}^{b}=LSTM\left(x_{t},h_{t+1}\right)$ and $h_{t}=\;\text{concatenate}\left(h_{t}^{f},h_{t}^{b}\right)$. Here $h_{t}^{f}$ and $h_{t}^{b}$ are representation for forward and backward pass. We use LSTM for each node (component/region) individually, sharing weights of LSTM among the nodes. As shown in Eq. (1), LSTM’s usually take a vector **x**_*t*_ as input at each step, however, we give $x_{t}^{i}$ (scalar value) as input to the LSTM along with hidden vector and receive $h_{t}^{i}$ for the node *i* at time-point *t*, which solves the window size problem occurring in dynamic-FNC studies. To make it easier to understand, one can assume that in our model the window size is 1. This allows us to later instantaneously compute connectivity matrix (links/edges) between the nodes at each time-point. The biLSTM receives temporal values of each component/region separately but share the weight matrices across regions. This allows the biLSTM to learn the temporal connections by looking at multiple nodes but does not learn spatial dependencies among nodes. For this exact reason we use self-attention across nodes.

#### 2.2.2. Self-Attention

A node in a graph can be linked with other nodes represented as the edge connectivity between them. The connectivity between nodes influence the value of a node $\left(x_{t}^{i}\right)$ at a certain time-point. Thus it is important to measure the connectivity between nodes for the construction and interpretation of the graph. In our fMRI data where each *x*^*i*^ is a brain region/component, capturing the DC or DNC between nodes shows how brain networks are linked with each other and the direction of flow of information between brain networks. The estimated matrices can then be used to explain brain working and brain disorders. Connectivity between brain regions is independent of the structural connectivity and thus is unknown. To capture the directed connectivity between brain regions, we use a self-attention module.

Self-attention module captures the weights between *n* inputs of a sequence. Since in a dynamic system (brain network), the connectivity between nodes can change at any instance, therefore, at each time-point *t* we pass a sequence of *n* vectors $h_{t}^{1}\ldots h_{t}^{n}$, *n* = total nodes, as input to the self-attention module and create the weight matrix **W**_*t*_, where each $W_{t}\in {\mathbb{R}}^{n*n}$ is the connectivity weight matrix of input nodes at time-point *t*.

The self-attention module creates three embeddings, namely, key (**k**), value (**v**), and query (**q**) and creates new embeddings for each input using these embeddings. The following set of equations can sum up the whole process. For simplicity, we omit the *t* from these equations. ^⊤^ represents transpose and ⊕ represents concatenation.


(2) $$k^{i}=h^{i⊤}W^{\left(k\right)},\;v^{i}=h^{i⊤}W^{\left(v\right)},\;q^{i}=h^{i⊤}W^{\left(q\right)}\;K={⊕}_{i=1}^{n}k^{i⊤},\;w^{i}=\mathrm{softmax}\left(q^{i}K\right)\;W={⊕}_{i=1}^{n}w^{i}$$

Here $W\in {\mathbb{R}}^{n*n}$ is the connectivity matrix between *n* nodes in the graph. As brain disorder are associated with disruptions in the connectivity of brain’s intrinsic network, we only use our learned directed connectivity matrices **W** for downstream classification and not the features, thus forcing the model to estimate the differences in connectivity between the two classification groups (e.g., HC and patients). As DICE is tuned to estimate the DC or DNC for the groups of subjects and output the it, DICE captures and shows the basis of downstream classification. The DC or DNC estimated by the model can be easily represented as a graph which are extremely easy to interpret. The self-attention glass-box layer shows task-dependant nodes (brain regions) and their connectivity.

The features that represent time-courses are used to learn/estimate the DC or DNC structure. As the true connectivity/graph structure is never available in many applications to directly compare with, we propose that a connectivity matrix leading to state-of-the-art classification performance makes it more reliable than using the representations/embeddings for classification.

#### 2.2.3. Temporal attention

As we use only the connectivity matrices learned by the model for downstream classification. For this purpose, we need to create a single weight matrix *W*^*f*^ based on the *W*_1−*T*_ matrices. For the downstream classification task, not all the time-points are equally important, hence it is crucial to incorporate a temporal attention module which assigns weight to each **W**_*t*_ and calculate a weighted average of all the weight matrices. We introduce a novel temporal attention module which we call global temporal attention (GTA).

##### GTA:

**Fig. 2.**
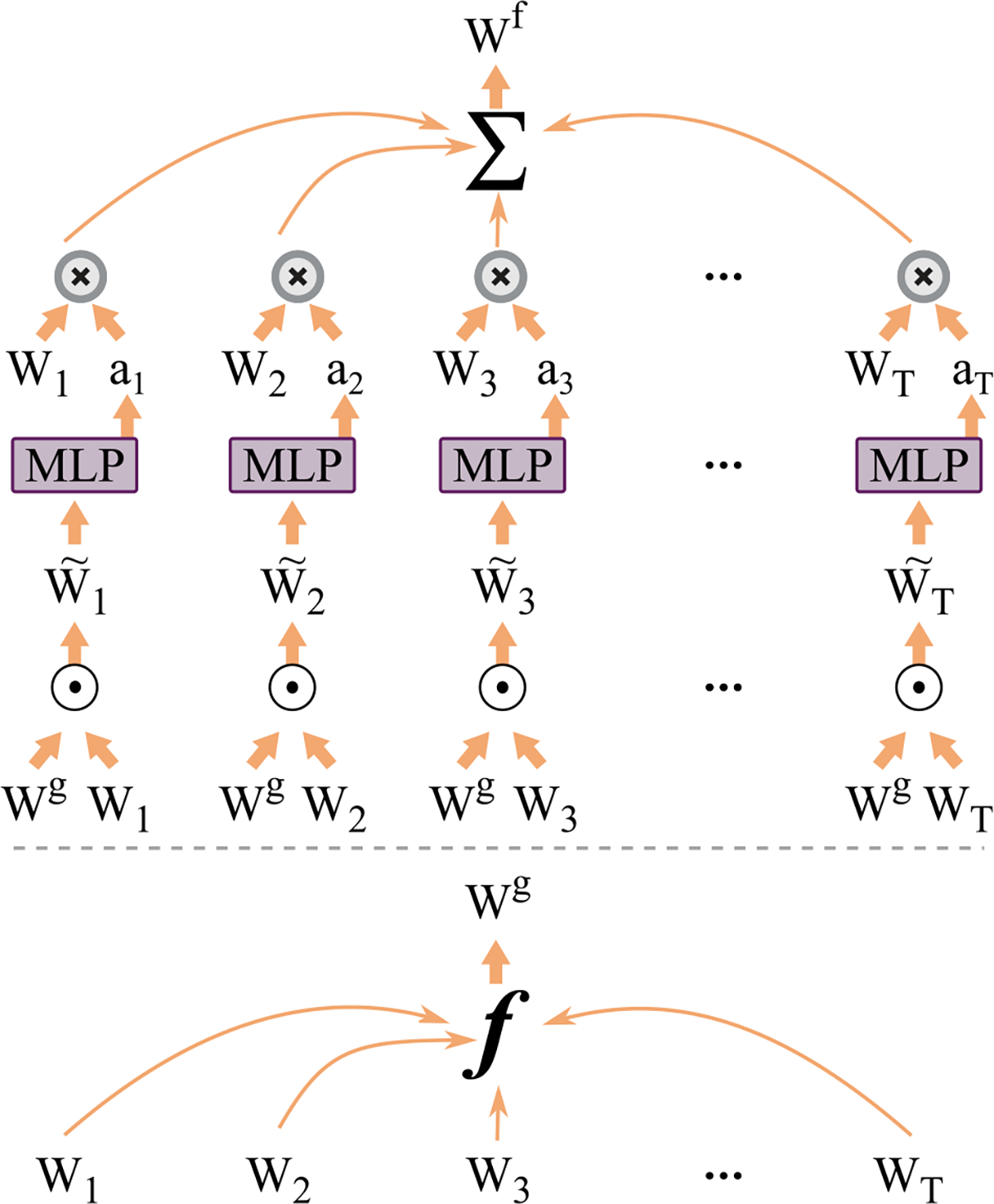
GTA architecture for temporal attention. **W**_1−*T*_ matrices are summed to create **W**^*global*.^ Using **W**^*global*^ and **W**_*i*_ attention score *α*_*i*_ is created for each time-point. Refer to equations in 3 and 4 for working details. Here ***f*** denotes the average function.

To give the attention module a global view of the graph, we present GTA. The global view allows the model to learn how each DC contributes to the global graph or structure of the data in the downstream task. We create an average of all the *T* DC and call it **W**^*global*^ representing the global view. We then compare the similarity of each local **W**_*t*_ with the global view and use them to create the temporal attention vector ***α***. Figure 2 shows the architecture details.


(3) $$W^{global}=\frac{1}{T}\sum_{t=1}^{T}W_{t}\;{\tilde{W}}_{t}=W_{t}⊙W^{global}\;\alpha =({⊕}_{t=1}^{T}\left(\left(\left(\left(\;\text{flat}\;\left({\tilde{W}}_{t}\right)\right)W^{MLP_{l1}}\right)W^{MLP_{l2}}\right)W^{MLP_{l3}}\right)$$

Here ⊙ is the Hadamard product (Million, 2007) between matrices. **W**^*f*^ is computed as:

(4) $$W^{f}=\sum_{t=1}^{T}W_{t}\alpha_{t}$$

### 2.3. Training

We used GTX 2080 with PyTorch as ML framework for our experiments. The hidden dimensions for the biLSTM was set to 100, whereas, self-attention including key, query, and value modules, were all set to 48. The dimensions of multi-layer perceptron (MLP) layers for calculating temporal attention vector were *η*_1_ * *len*(*flat*(**W**_*t*_)), *η*_2_ * *len*(*flat*(**W**_*t*_)), and 1 with *η*_1_ = *η*_2_ = 0.05. We noticed in our experiments that multiple heads of self-attention increases stability of the estimated DC. We used batch normalization after the first MLP layer. ReLU activation was used in our model between the MLP layers. A final two-layer MLP was used to get logits for binary classification problem with **W**^*f*^ as input with dimensions 64 and 2. We used cross-entropy loss with Adam optimizer. Let *θ* represent the parameters of the entire architecture, $\hat{y}$ being the predictions and *y* the true labels, the loss is calculated as:

(5) $$loss\;=\;\text{CrossEntropy}\;(\hat{y},y)+\lambda \|\theta {\|}_{1}$$

(6) $$\theta^{*}=\arg\min_{\theta }(loss)$$

We also experimented with additional loss terms to encourage the model to estimate connectivity matrices where the values of the main diagonal are closer to 1. Please refer to Section Appendix B for details. We used L1-regularization to get a sparser solution. *λ* (regularization weight) was set as 1*e*^−6^ and learning rate was 2*e*^−4^. Based on the experiment, we reduced the learning rate either when validation loss reached plateau by a factor of 0.5 or exponentially with *γ* = 0.99. Early stopping was used to stop training the model based on validation loss and patience of 25. For each dataset (ICA components or ROIs), to have a fair result, we perform n-fold testing where the value of n depended on the dataset and methods we compared against. For each test fold we performed experiments with 10 randomly-seeded trials. We report the mean AUC-ROC (Area Under Curve - Receiver Operating Characteristic) across the n test folds and the 10 randomly-seeded trials as it is a more reliable metric than simple accuracy for binary classification tasks. For example, for FBIRN data we had 18 test folds and for each fold we performed 10 trials, which gives us a list of 180 AUC-ROC values and we report the average of these values. In some cases we also report other metrics as well, such as accuracy. Due to the size of the data, we made some hyper-parameter changes for HCP region-based (ROIs) experiments. The hidden dimension size for bilstm and self-attention module was set to 64 and 32. *η*_1_ was set to 0.005. Furthermore, because of memory constraints encountered during HCP region experiments, during both training and testing we divide the total time-points (1200) into a set of three, each having 400 time-points. We create logits for all and compute the mean to get final logits. Batch size was set to 32.

#### 2.3.1. Hyper-parameters selection and fine-tuning

All the parameters (hidden dimensions, number of layers, *η*_1_, *η*_2_, *λ*, learning rate, *γ*, patience, batch size) mentioned in Section 2.3 were set as hyper-parameters. We fine-tuned these hyper-parameters based on the average performance of the model on validation dataset across all the folds. We did not perform hyper-parameters tuning based on the test folds and we report only test-set results. We also want to note here that we permuted the order of subjects for each dataset and performed the experiments using the permuted order. This was done to avoid imbalance of subjects in the folds. On the same lines, when dividing the data into n-folds (test folds) we tried to balance the number of subjects of both classes in each fold. For example, in case of FBIRN data with 311 subjects and 151 and 160 subjects in class 0 and 1 respectively. When performing 18 fold testing, each test fold consisted of $\left\lfloor \frac{151}{18}\right\rceil$ subjects from class 0 and $\left\lfloor \frac{160}{18}\right\rceil$ subjects from class 1 and the rest of the data was used for training and validation, where we kept the validation set size same as the test set size. The validation set was used for hyper-parameters tuning, early stopping during training and selecting the model to apply on the test data. We made sure that no subject (or sessions of a subject) repeated across training, validation and test sets. The exact size of training, validation and test set can be calculated using the criteria mentioned above and the total number of subjects and number of folds mentioned in Table 1. In some of the experiments keeping the same number of subjects in each fold created a small data leakage at the end. For the results reported, the maximum leakage was for FBIRN dataset with 18 test folds. For this purpose, we performed another experiment on FBIRN dataset where the last fold had all the left out subjects to prevent any data leakage. This had no effect on the performance of the model. Refer to Table A.11 for results.

## 3. Experiments

To test if DICE accomplishes all the goals, we perform detailed experiments by classifying three brain disorders, classify male and female groups for HCP and OASIS subjects, and predict age for OASIS subjects. We perform experiments for all datasets using ICA time-courses and perform experiments on FBIRN, ABIDE and HCP data using regions-based (ROIs) data as well. In this paper we refer to matrices capturing functional connectivity between networks at a whole-brain level as functional network connectivity (FNC) (Allen et al., 2011b; Jafri et al., 2008) and when operating on ROIs – as FC. We report the average results for all the trials. Depending on the experiment, we compare our classification results with state-of-the-art DL methods (Arslan et al., 2018; Gadgil et al., 2021; Kim and Ye, 2020; Mahmood et al., 2021; 2019; 2020; Weis et al., 2019; Zhang et al., 2018a) and ML methods (Support Vector Machine (SVM), Logistic Regression (LR)). To avoid any discrepancy we report the results of the DL methods directly from the published studies, even though some studies use test data instead of validation data for selecting the best performing model/parameters. For ML methods we used the python package Polyssifier^5^ which selects the best model/parameters based on the performance on validation data.

To show the efficacy of our model, we divide our results into three broad categories. In the following sections we show a) classification performance of our model, b) learned DC and DNC and c) the effects of temporal attention module.

### 3.1. Classification

**Fig. 3.**
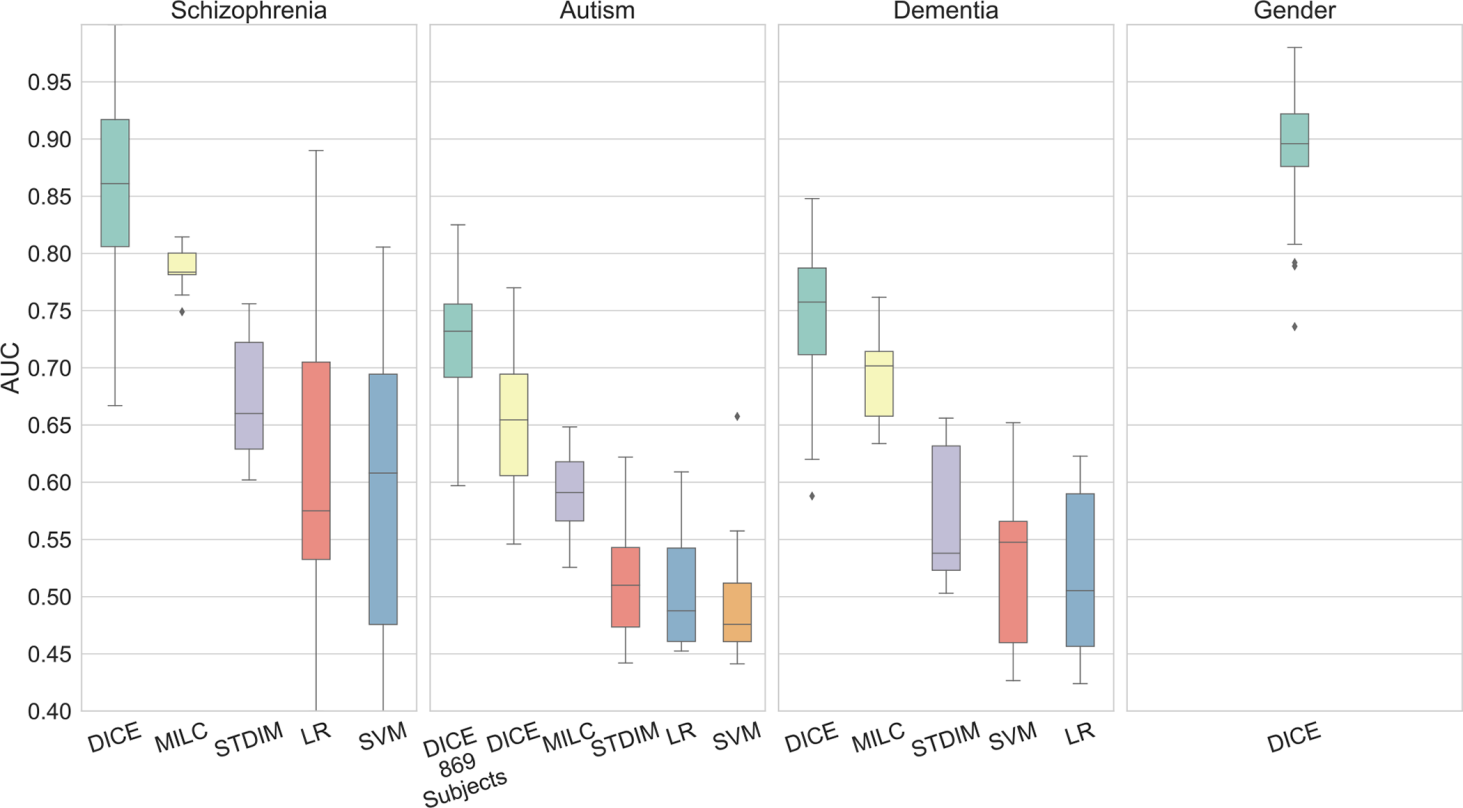
AUC comparision of DICE model with four different methods (MILC Mahmood et al. (2020), STDIM Mahmood et al. (2019), logistic regression (LR), support vector machine (SVM)), over four different datasets using ICA time-courses (Ref to Section 2.1.1). Our method significantly outperforms SOTA methods. We performed Autism experiments with 869 subjects (all TRs) as well. As we do not have a pre-training step we compare with not-pre-trained (NPT) version of MILC and STDIM. Input to ML methods were the same ICA time-courses, not the FNC matrices. We did not find any notable studies for gender classification of HCP subjects using ICA components as notable methods used ROIs based data. We compare the results using ROIs in Table 2.

Figure 3 shows the classification performance of our model using ICA data, Table 2 shows the performance using region-based (ROIs) data of FBIRN and HCP, and Table 3 shows results on ABIDE region-based (ROIs) data.

Our model beats every state-of-the-art method used for comparison in this study in almost every metric for both ICA and region-based (ROIs) fMRI data across all datasets when using similar input data (fMRI). As our model does not use phenotypic information about subjects, it lacks behind (Cao et al., 2021; Parisot et al., 2018) on ABIDE. Parisot et al. (2018) reports a decrease of ~ 2.5 AUC by using a different phenotypic information which clearly shows the dependence on phenotypic data. Whereas, Ktena et al. (2018) reports much lower AUC score by using only fMRI data. ML methods fail completely even on ICA data, We attribute this failure to two reasons. 1) The number of dimensions (*m*) being much higher than the number of subjects (*n*), thus creating the curse of dimensionality (*m* >> *n*) and 2) The ML methods do not compute a graph structure for estimating the connectivity between the networks/components and instead mostly work with independent networks/components. According to our knowledge, no other model gives such high classification score across four neuroimaging datasets. The high classification score of the model computed using only the learned DC structure increases the confidence in the correctness of the learned DC structures.

### 3.2. Directed connectivity

The learned interpretable, task-dependent (flexible) directed connectivity structures by our model is the most important contribution of our work. As this is a novel work, we show in detail, different aspects of the learned connectivity structures. We a) compare our learned DNC with FNC computed via PCC, b) compare the differences in DC and DNC between multiple classification groups, c) show how direction matters in connectivity, something which is not captured by FC and FNC, d) dive into the fact mentioned in introduction that unlike computed FNC (using PCC) our learned DNC is task dependent and changes based on the downstream task (ground-truth signal) and e) show the dynamic connectivity states for FBIRN data for HC and schizophrenia (SZ) subjects. All the aspects (a-e) discussed in detail in following sections show the correctness and interpretability of the learned DC and DNC. The interpretability of the connectivity matrices estimated by our model give insight into how brain networks are linked with each other and with the downstream classification task. This is very crucial to understand brain disorders and relevant brain networks. Unlike typical FC and FNC which ranges from −1 to 1, our learned matrices are based on attention and hence ranges from 0 to 1. More information on this in Appendix B.

#### 3.2.1. DNC vs FNC

**Fig. 4.**
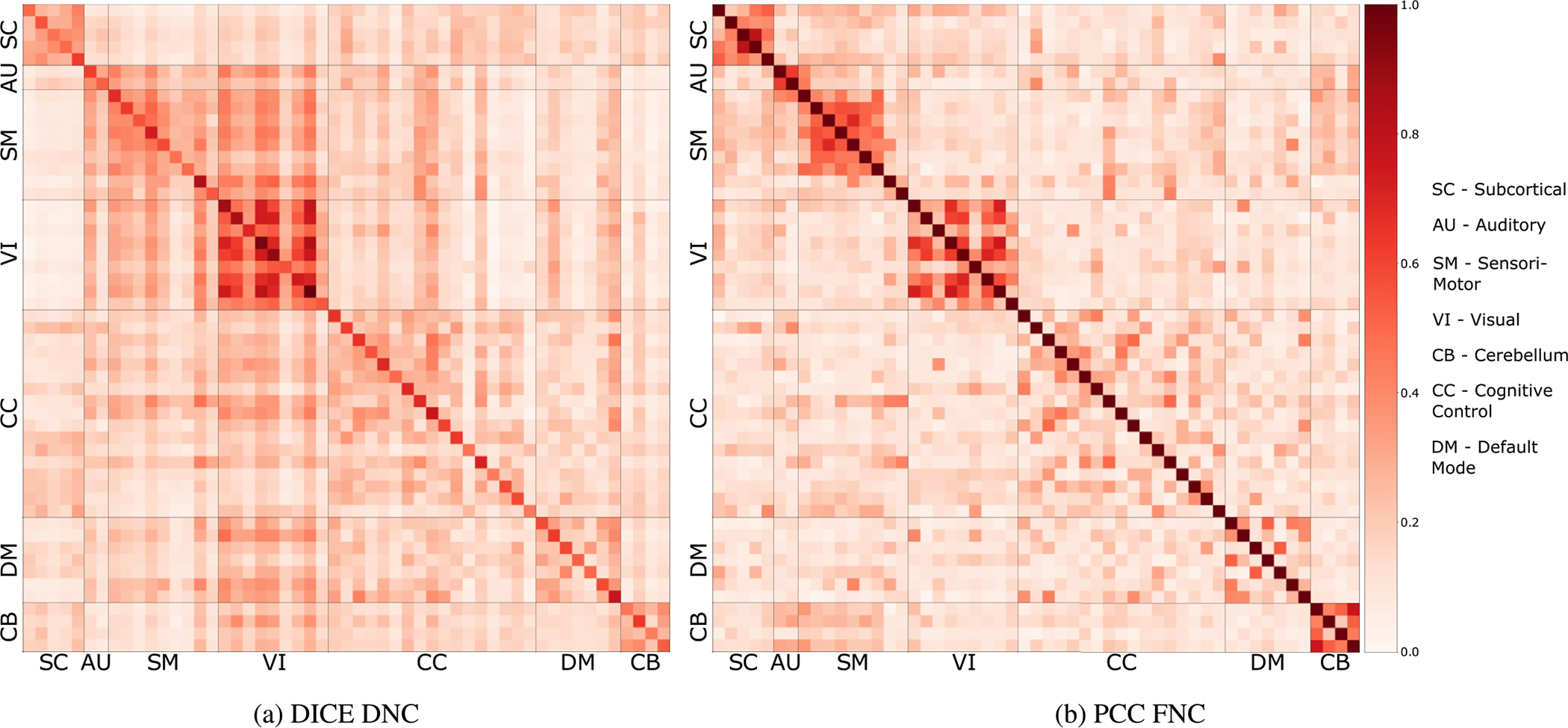
We compare our estimated DNC with computed FNC using PCC method. 4 a is the connectivity matrix generated by our model for FBIRN dataset. We used a test fold of 16 subjects and computed mean FNC for all subjects (10 trials per subject). 4 b is the mean connectivity matrix of the same subjects generated by PCC. Both figures show similar intra-network connectivity patterns, which verifies the correctness of the connectivity matrix learned by our model. Our estimated DC is directed and captures more inter-network connectivity than FNC. To match the positive weights of our model, we have normalized the FNC from 0 to 1 instead of −1 to 1.

As the true connectivity between brain networks is not known, we compare our learned DNC with FNC. Figure 4 shows the DNC learned by our model and the FNC computed using PCC using ICA components for FBIRN dataset. The DNC is **W**^*f*^ explained in Section 2.2.3. Both DNC and FNC is the mean matrix for highest performing fold of FBIRN dataset with 16 subjects. The 100 ICA components are divided into informative (53) and noise (47). We show the connectvity between 53 non-noise components. These components are further divided into 7 domains/networks following (Allen et al., 2011a). Both matrices clearly show high intra-domain connectivity. The learned DNC shows similar pattern of FNC which increases the confidence in the DNC learned by our model but there are very important differences between the two. **Inter-network connectivity:** We see that our estimated DNC finds much more inter-network connectivities than the FNC which is mostly intra-network and has very low scores between networks. **Directionality:** Regarding the direct influence, DNC estimated by our model is directed and shows components in visual affecting components through out the domains, such information is not present in the FNC which is un-directed (symmetric across main diagonal) and does not show the direction of connectivity. Refer to Section 3.2.2 for more detail on this.

To compare the connectivity matrices in terms of classification results, we use an LR model and perform classification by first training and testing the model using PCC-based FNC and then by our estimated DNC as input. Refer to Table 4 for comparison.

#### 3.2.2. Directed connectome

**Fig. 5.**
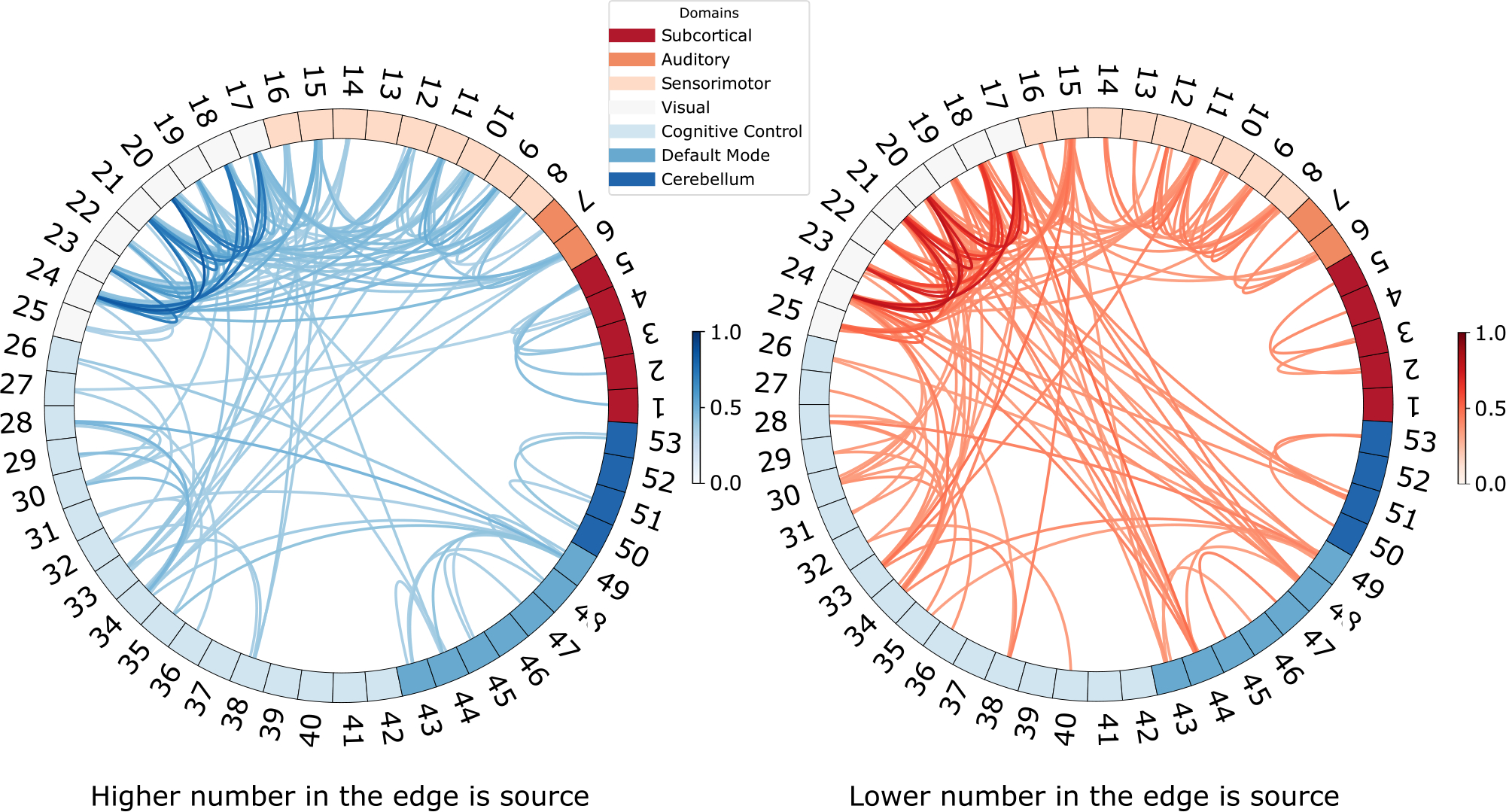
We show the top 10% directed edges of FBIRN DNC. The numbers represent the 53 non-artifact components. The figure clearly shows the high intra-domain connectivity which matches the existing literature. Direction clearly matters as visual components affect other components but not the opposite way. The direction of edges between CC and SM networks is also of significance.

Capturing directed connectivity is one of the methods to understand the direction and flow of information in the brain. Learning the direction of connectivity is one of the main advantages of our model as it might explain the direct influence of brain networks upon each other. To show the direction between components, we divide the DNC of FBIRN subjects into two connectomes showing the direction. Figure 5 left shows the edges from *a* to *b* where *a* > *b*. For example the edge between (8,23) shows the edge from 23 to 8, whereas, Fig. 5 right shows the opposite. It is clear from the figure that direction matters and the connectivity between brain regions is beyond simple statistical dependence. For example, Fig. 5 shows that the components in visual network (VIN) affect components in other networks and the edges in the opposite direction are relatively much fewer. We also see direction of connectivity from cognitive control (CC) to sensorimotor (SM). Existing studies (Breukelaar et al., 2017; Cole and Schneider, 2007; Tsai et al., 2019) show that cognitive control is responsible for activities like attention, remembering and execution, things which are required when doing a motor task controlled by sensorimotor. Such directionality is important to study brain’s working in more detail and is not present in FNC used by existing methods. The results are further discussed in Section 4.1

#### 3.2.3. Connectivity differences among groups

**Fig. 6.**
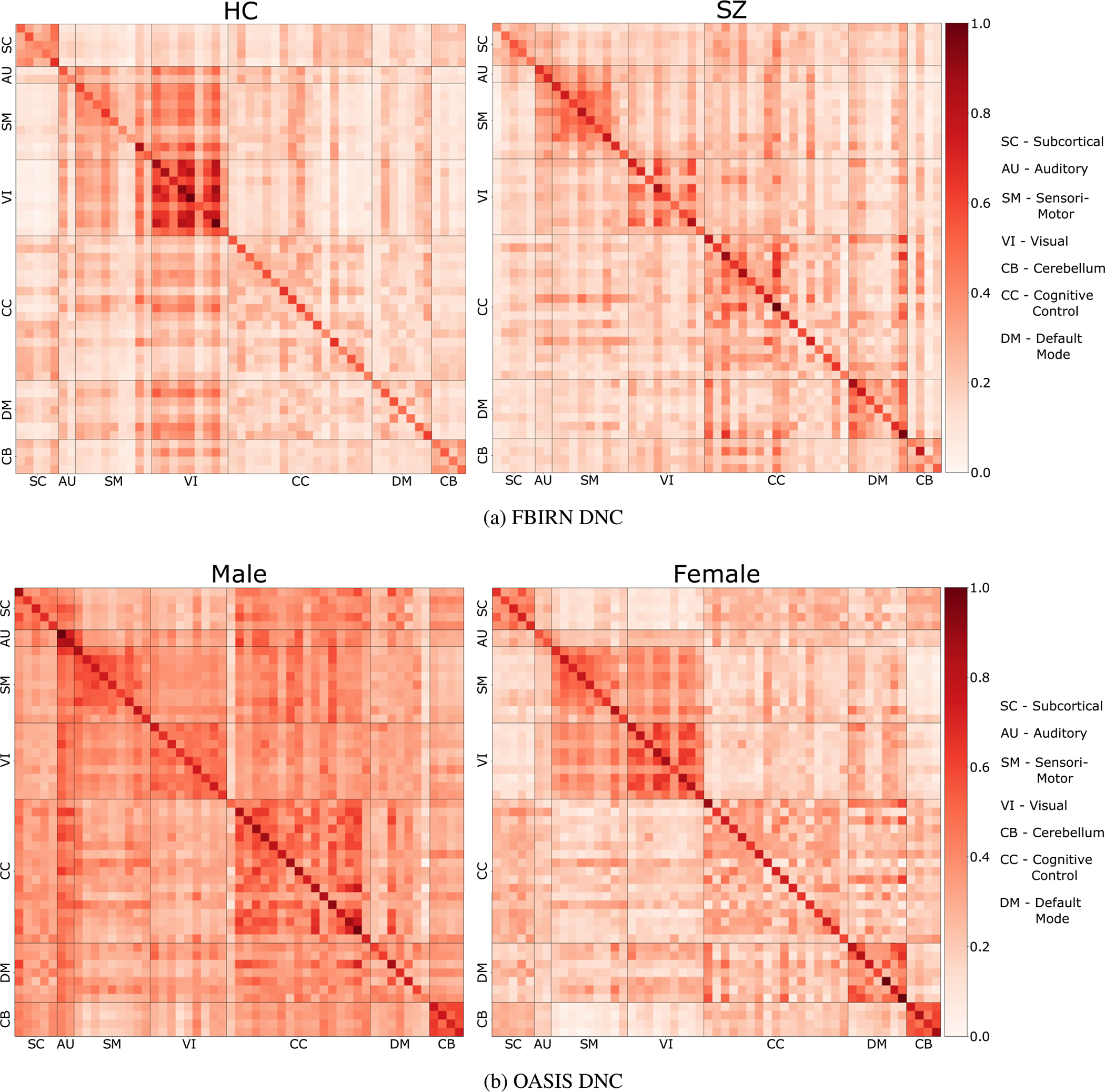
We compare the estimated DNC across the binary classification groups using ICA data. Figure 6a is the estimated DNC on FBIRN data for HC and SZ patients. We see high inter and intra-connectivity in SM and VI networks for HC, which is missing in SZ patients. Figure 6b compares DNC between male and female groups using OASIS data. Female group shows hyper-connectivity in DMN and hypo-connectivity in SMN when comparing to male groups.

As hypothesized that brain disorders are linked with the connectivity of brain’s intrinsic networks, we show how the learned DC and DNC changes for subjects belonging to different groups. Figure 6a shows the DNC estimated by our model of HC and SZ subjects for FBIRN data whereas Fig. 6b shows DNC of male and female groups for OASIS dataset. Both results are computed using ICA pre-processed data. For ICA based DNC, there are similarity between the two matrices as they come from the same joint ICA. However, there are visible difference between the two for multiple networks like visual (VI), cognitive control (CC), default-mode (DM) and cerebellum (CB). The biggest difference between HC and SZ groups seems to be in the connectivity strength for VIN. For OASIS results 6 b we see that females show high connectivity scores in default-mode network (DMN) compare to males and low sensori-motor network (SMN) connectivity compare to males, this has been verified by existing studies (Filippi et al., 2013; Kim et al., 2021; Mak et al., 2016; Ritchie et al., 2018). To verify this by numbers, we use statistical testing to compare the two groups (male, female) and compare average connectivity for male and female in DMN and SMN. Table 5 shows the statistical results.

**Fig. 7.**
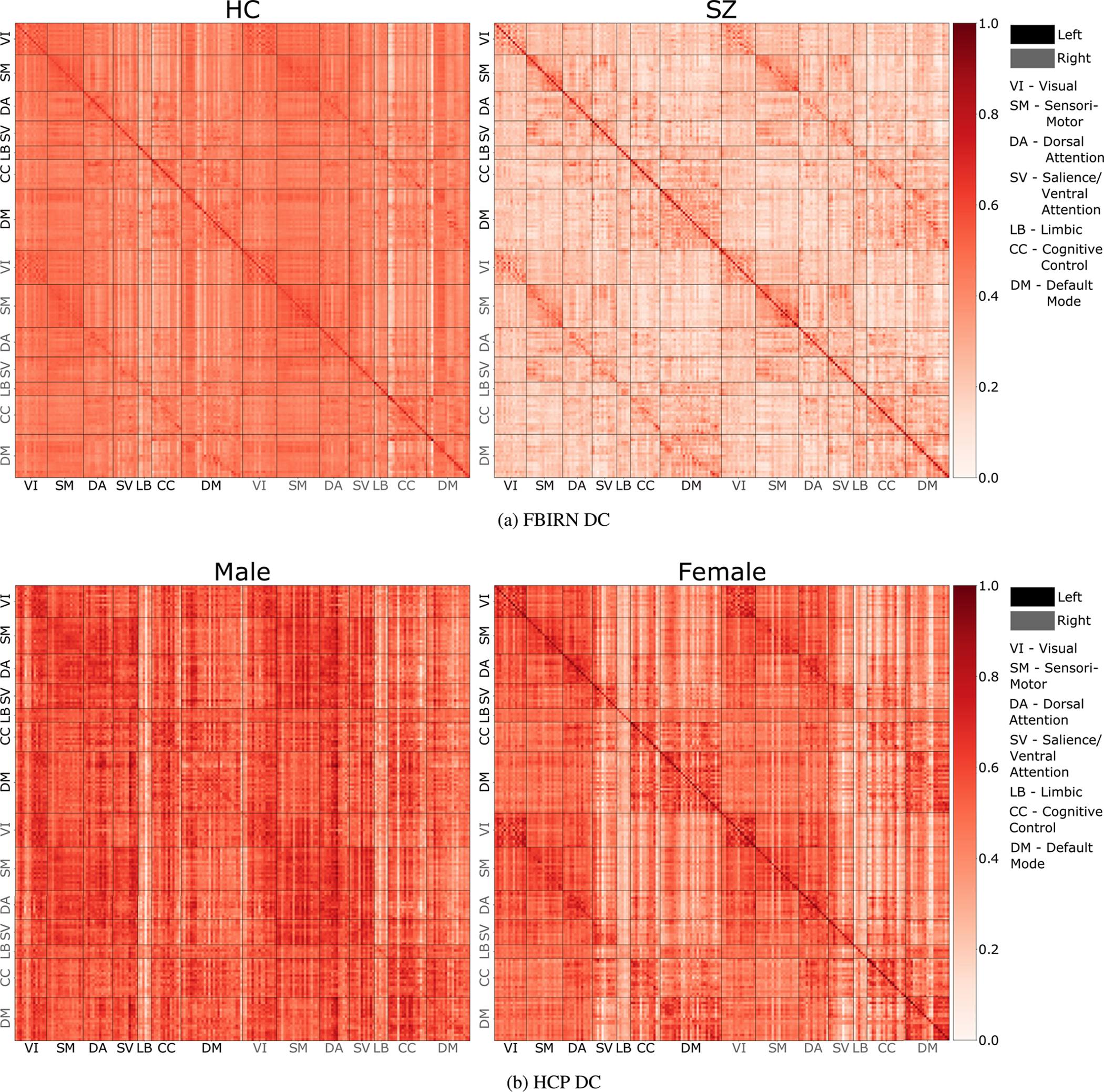
We compare the estimated DCs of HC with SZ and male with female using region-based (ROIs) FBIRN and HCP data. 7 a show high weakly connected brain networks for SZ subjects whereas 7 b show hyper-connectivity of DMN and hypo-connectivity for SMN for females as compared to females. The black and grey color denotes the regions in left and right side of the brain. Refer to Table 7 for a statistical comparison between female and male DCs.

Figure 7 performs the same experiment for region-based (ROIs) data. Here the regions for both sides of the brain (left and right) are divided into 7 domains following shaefer (Schaefer et al., 2017). Again, in Fig. 7b for HC we see high connectivity score between regions of the same network. We also see connectivity between regions of same network across left and right side of the brain. The diagonals on top and bottom of the main diagonal shows this. Whereas the DC of SZ subjects is weakly connected compared to HC and is mostly shows intra-network connectivity. The sparsity explains and support the existing literature explaining SZ as functional dysconnectivity between brain networks (Culbreth et al., 2021; Lynall et al., 2010; Morgan et al., 2020; van den Heuvel et al., 2010; Yu et al., 2011; Zhang et al., 2019; Zhu et al., 2020).

Figure 7 b compares male and female groups based on region-based (ROIs) HCP data. We see similar patterns of hyper-connectivity of DMN and hypo-connectivity of SMN in females as compared to males. As the region-based (ROIs) parcellation divides the brain into left and right, we also see that females have high intra-network connectivity between left and right side of the brain as compared to males.

To verify the visual results, we use statistical testing to compare the DMN and SMN between males and females. The stats confirm the visual results with 1) female DMN showing higher connectivity than female SMN and male DMN, and 2) male SMN showing higher connectivity than male DMN and female SMN. We also see that the networks are highly statistically different. Refer to Table 7.

#### 3.2.4. Task dependent DNC

**Fig. 8.**
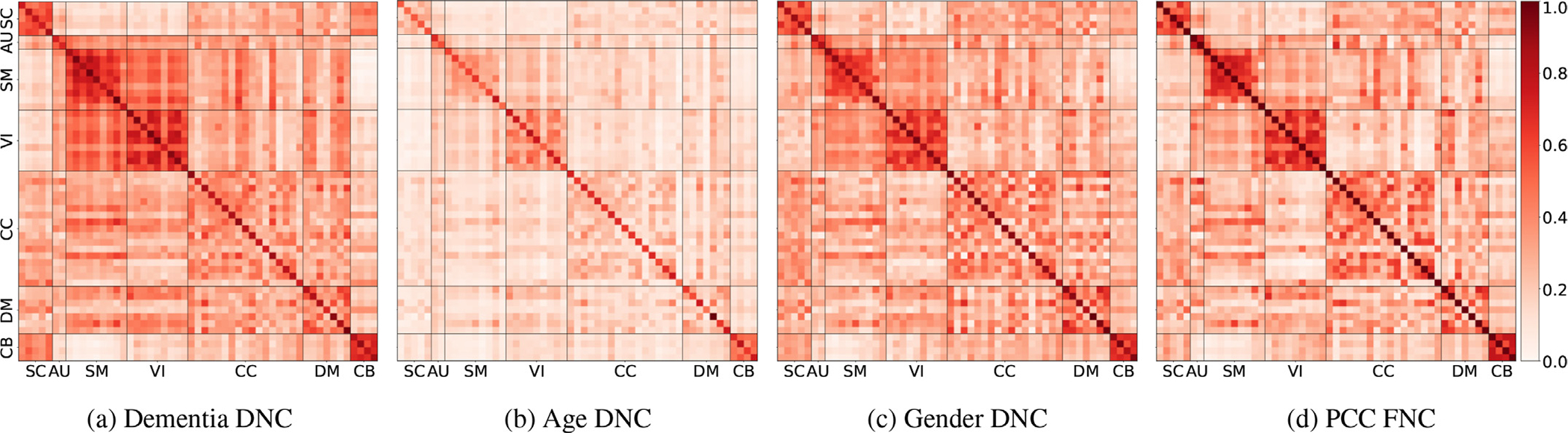
We show how our model estimates flexible DNC structures based on the ground-truth signal. We train our model for different classification tasks and use same test subjects to compare the estimated DNC for the subjects. All figures are mean DNC estimated for the same subjects with 5 randomly-seeded trials. 8 a is the mean connectivity matrix estimated by our model when trained to classify dementia. We see high connectivity values for SC, SM, and CB networks. 8 c is the mean DNC for the same subjects when the model is trained for gender prediction. We notice lower SM network connectivity and higher connectivity for DM network when predicting gender of OASIS subjects. 8 d is the FNC computed using PCC. The FNC is independent of the task and would remain fixed (inflexible).

Human brain can be divided into multiple parts/regions where each region is linked with a set of tasks. For example, the hippocampus is associated with memory. Thus it is important to know which region/network(s) are linked with the downstream task (e.g. disorder classification). Finding the linked regions/networks would help us understand the disorder and allow to study the association of these regions/network(s) with the disorder in more detail. In this section, we see how the DNC structure learned by our model changes and identifies different networks for the same subjects based on the downstream task. For this purpose, we perform an experiment, where we compare the estimated DNC for OASIS data when predicting dementia, age and gender of the same subjects. The number of subjects were balanced with both HC and patients equalling 50% of the total subjects but had ~ 62% female subjects. Figure 8 shows that our model produces task dependent DNC and the networks/domains showing high connectivity for each task adheres to the existing literature. The Fig. 8a shows the DNC learned when classifying subjects for dementia. We see high connectivity for components in the SM, DM, and CB networks. These networks are linked with dementia in existing literature, which support the results of our method. Whereas when classifying gender of same subjects, the estimated DNC is different and show high connectivity for components in DM and reduced connectivity for SMN. Figure 8d shows the FNC computed via PCC for the same subjects. As FNC computed using PCC is only data dependent, the FNC would remain same for all the tasks and shows the inflexibility of the method. Figure 8 therefore shows a) our model learns task dependent DNC and b) our model accurately finds networks linked with the downstream classification task. We see this as a significant advantage over studies which compute a fixed/static FNC using PCC and hence is independent of the downstream task. We see that Fig. 8b which is the learned connectivity structure when predicting age does not show high connectivity between networks and the connectivity values for SMN and DMN are almost same. This could be a reason of small age variance in the dataset.

We use statistical scores to verify the visual results. Table 8 shows the statistical difference between the three DCs as a whole and between DMN and SMN. We also compare the estimated DCs with FC 8 d.

We see that all three DNCs are extremely statistically different. It is also proven that DMN is given higher connectivity scores for gender prediction whereas, SMN connectivity is much higher when predicting dementia comparing to gender and age prediction tasks. To clear how the connectivity values change for DMN and SMN we point out the average connectivity scores of the networks for dementia and gender classification and compare it with the values of DMN and SMN computed via PCC. The connectivity values in FC for SMN and DMN are 0.580 and 0.487 respectively (and would remain same irrespective of the classification task). Whereas, when classifying dementia our model show much higher SMN average value of 0.64 and a little decreased value of 0.478 for DMN showing a focus on SMN despite having more female subjects in the test set. When predicting gender for the same subjects the DNC estimated by our model has a decreased SMN value of 0.555 and increased value of 0.527 for DMN hence focusing less on SMN and more on DMN when compared to the dementia classifying task thus verifying that our estimated DCs are task-dependent and not only data dependent. We discuss the meaning and significance of this result in Section 4.3.

**Fig. 9.**
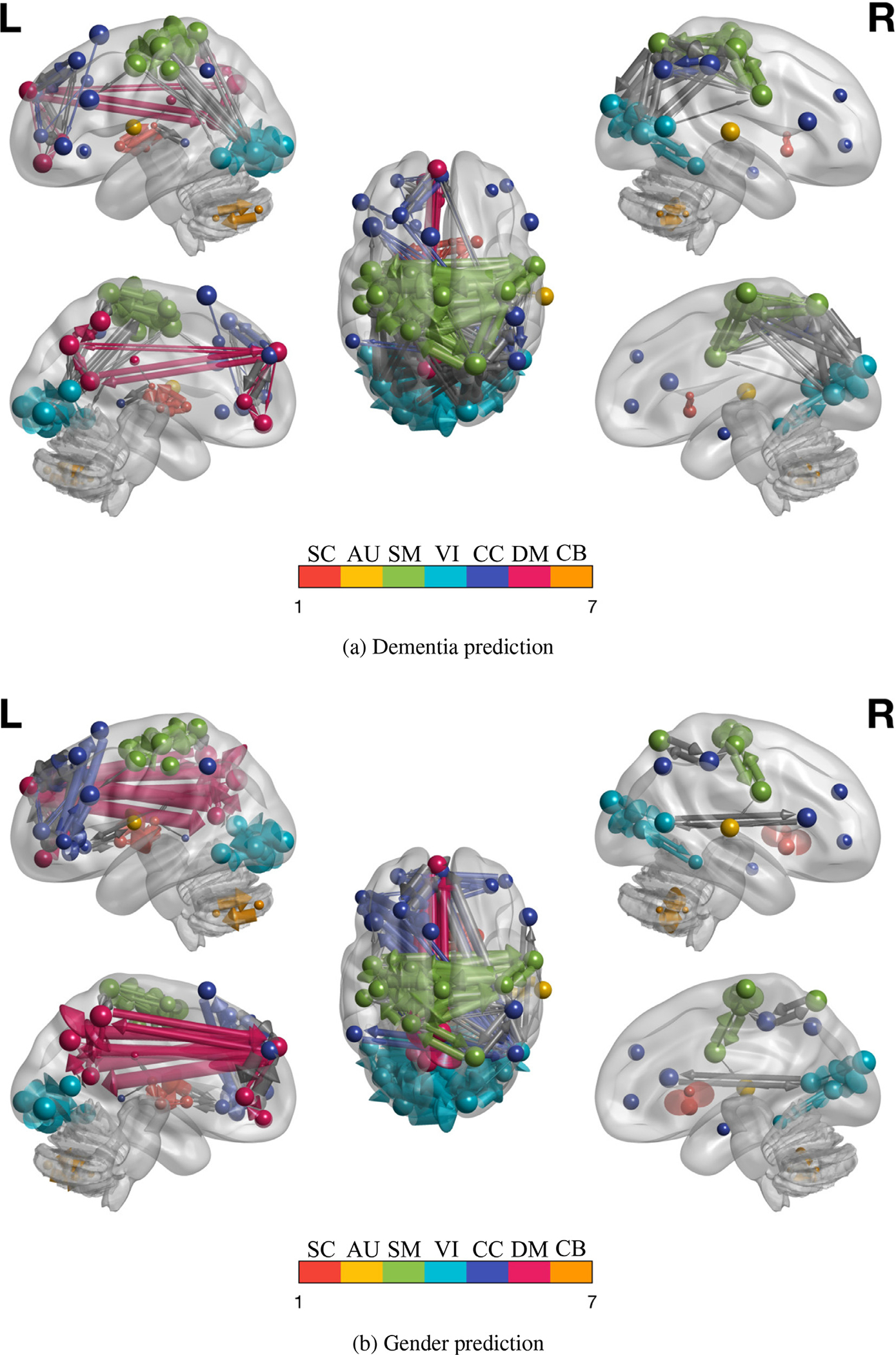
We map on the brain, the nodes and top 10% edges of the DCs, estimated for dementia and gender classification tasks, performed on OASIS dataset (same subjects). The size of the nodes is the sum of the outgoing and incoming edge weights. The arrows shows the direction of connectivity. We see a high number and size of nodes and edges for SMN and VIN for dementia 9 a, whereas for gender 9 b we see high node and edge size for DMN. Compare the red (DM) nodes and edges in Fig. 9a with b in the left side figures. Figure 9a also shows high connectivity between SM and VI networks which is missing in Fig. 9b (right side figures). This reveals the networks and edges (graphs and subgraphs) relevant to the classification signal (e.g disorder) without need of comparison with other data. The results and their impact are further discussed in Section 4.3. (For interpretation of the references to colour in this figure legend, the reader is referred to the web version of this article.)

To see the matrices as graph of nodes (regions) and edges (connectivity), we plot Fig. 8a and c on the brain and show the results in Fig. 9. The figure shows high number of nodes and edges among components of VIN and SMN and among the two networks for dementia classification 9 a, and high number of nodes and edges among components in DMN for gender classification 9 b.

#### 3.2.5. Dynamic connectivity states

Studies like (Allen et al., 2012; Calhoun et al., 2014; Hutchison et al., 2013; Sakoğlu et al., 2010) show that human’s brain FC is dynamic and can be used to find patterns which are not visible in static FC studies. These studies show that dynamic FC show re-occuring patterns. To study these patterns, dynamic connectivity of the human brain is divided into distinct *k* states (Damaraju et al., 2014; Fu et al., 2021; Rashid et al., 2014). There are multiple methods proposed to find the *k* states with k-means being one of the most used methods. These studies show that the transition and time spent in each state is different for patients (SZ, dementia, autism) and HC. To validate our results and to find such patterns we use k-means to find *k* (5) such states using the DCs estimated by DICE for FBIRN dataset. We calculate and compare the time spent by both groups (SZ and HC) per state.

**Fig. 10.**
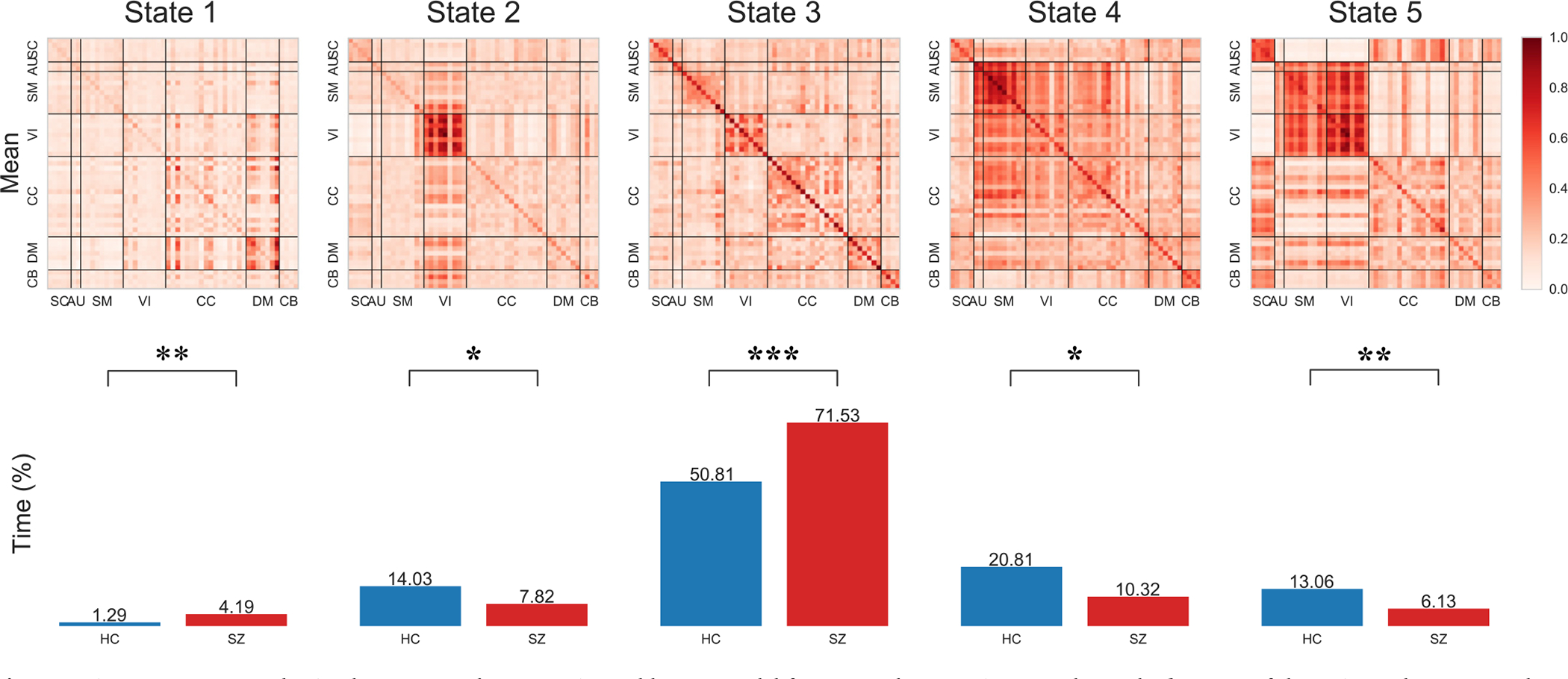
Five states computed using k-means on the DCs estimated by our model for FBIRN dataset. First row shows the *k* means of the estimated DCs, second row shows the percentage time spent by both groups in each state, with the total time points being 155. Time spent in each state by SZ and HC differ significantly and matches the existing literature. We see that a) time spent in each state is different by HC and SZ, b) SZ spend much more time in state 3 (weakly connected) than HC, c) HC spend more time than SZ in states (2,4, 5) which show high connectivity for VI, and SM networks, and d) Standard deviation of time for SZ is much higher (320.47) than HC (206.26) which shows that SZ stay in one state much more than HC which tend to change state more often. The stars denote the significance of difference in time spent in each state by the two groups. Table 6 shows the *p*-value significance ranges.

**Fig. 11.**
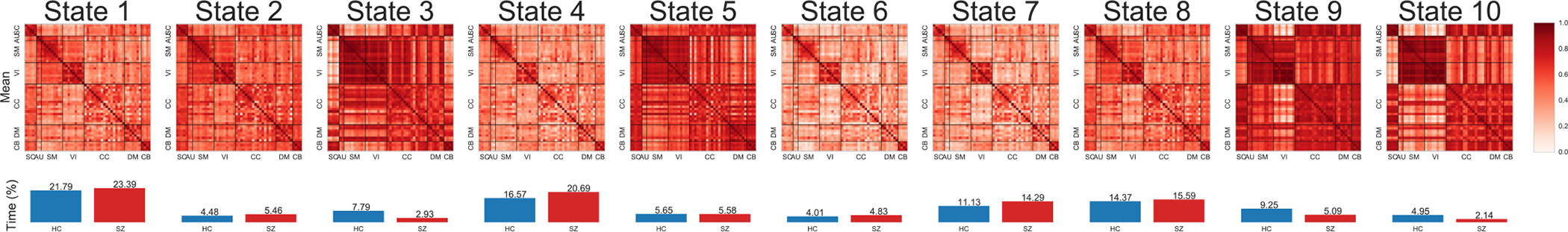
We show *10* states captured by k-means on the temporal DCs estimated by DICE on FBIRN complete dataset. The rows shows the means and the percentage of time spent by HC and SZ subjects in each state. We see that DICE can capture more states than the standard (4–5) states captured by window-based approaches. The additional states not present in Fig. 10 show the change of direction in connectivity. State 9 shows the opposite direction of connectivity between VIN and other networks, where VIN has mostly incoming edges. The ratio of time spent by HC and SZ subject in different states is similar to the results of Fig. 10.

Figure 10 shows that SZ subjects spend more time in weakly connected states (1,3) than HC which stay in states which show high connectivity score for visual (VI) and sensorimotor (SM). We also see that HC tend to change state more often than SZ which spend ~ 66% time in one state (number 3). Existing studies (Miller and Calhoun, 2020a; 2020b; Yaesoubi et al., 2018) show that window-less approach can find dynamic patterns that are not captured by the vastly used window-based approach. As DICE is an instantaneous model, we investigate if DICE can capture more dynamic states than the window-based dynamic-FNC studies. For this purpose, using elbow method (Marutho et al., 2018), we found that the best *k* for the estimated DCs is not 5, and set *k* = 10 and show the resultant states in Fig. 11. We see the model captures additional states that were not visible with *k* = 5. The additional states found show the pattern of directionality, specially in the states where HC spend more time than SZ. For example, in Fig. 10, state 2 show dense connectivity for components in VIN and the direction is from VI to other states, and state 5 show similar direction but with sparse connectivity. Figure 11 captures the additional state (9) which shows the opposite direction, that is, VIN has mostly incoming edges. We believe this state represents the brain activity when different networks (e.g. SMN) are giving input to VIN to control the vision. We discuss this result in Section 4.4.

### 3.3. Temporal attention

**Fig. 12.**
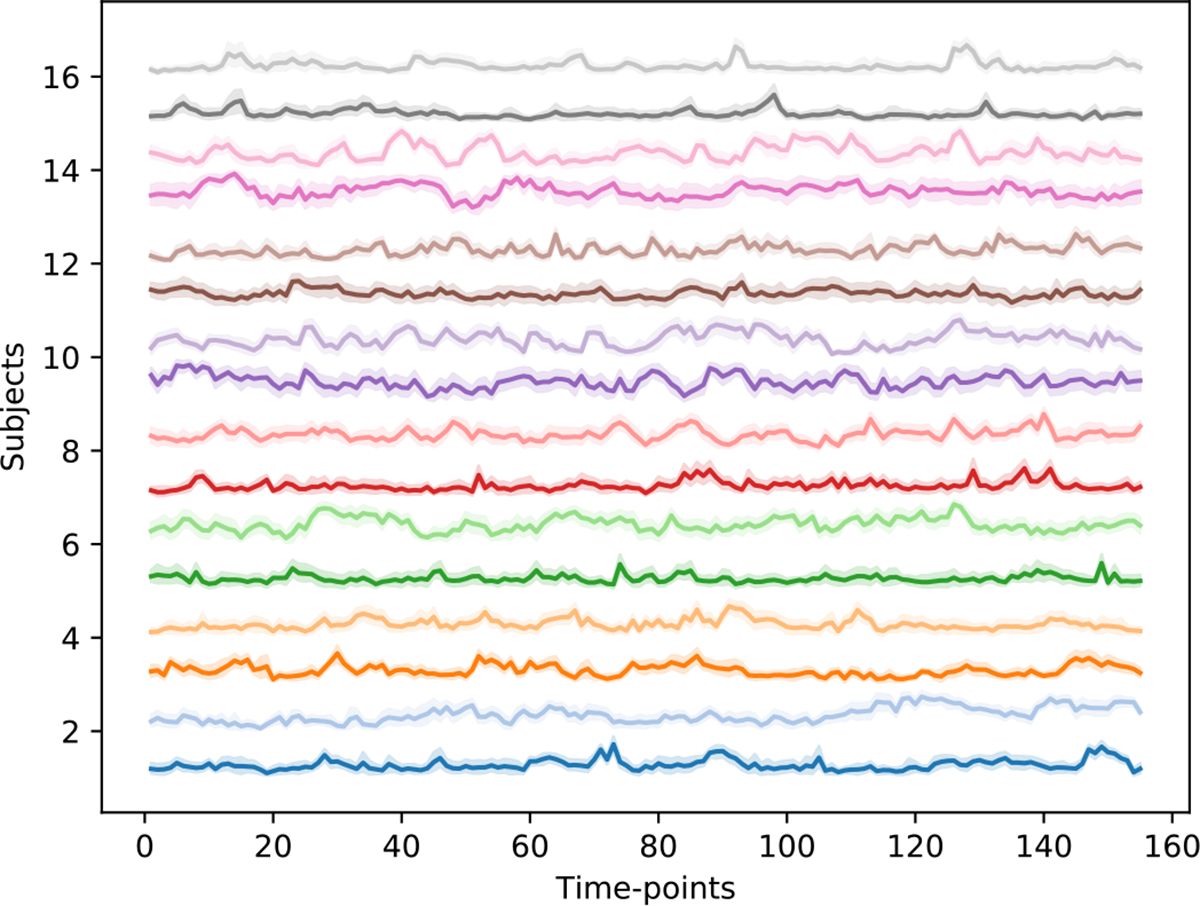
Temporal Attention weights for one of the test folds (16 subjects) of FBIRN. Attention weights are computed using GTA module. X and y axis represent time-points and subject number respectively. We show that for each subject, the attention weights remain stable across multiple randomly-seeded trials (10). The values of the 10 trials are used to create the confidence interval for each subject. The consistency is greatly increased with an increase in number of training subjects. *Note:* For each subject we added the subject number to the attention weights to separate the weights, as for each subject the weights have a range of 0 – 1. Dark and light colors represent SZ and HC subjects respectively.

Our temporal attention module finds the important time-points that are relevant for the downstream task (e.g. gender prediction). As not all time-points are equally important for the downstream task, and fMRI data has low temporal resolution, the temporal attention is an effective way of finding important bio-markers for neuroimaging dataset. Finding the relevant time-points can help reduce the data and allow to focus on activities at specific points. Figure 12 shows the weights assigned to the subjects of FBIRN.

We show weights for 16 subjects (8 per class) with 10 randomly-seeded trials. The results show that the temporal attention module is very stable and assign similar weights to the time-points for every trial.

To further check the correctness of the time-points selected by our model and how these time-points are useful in terms of classification performance, we perform an experiment where after training the model, we use **W**_*t*_ of the top 5% values to train an LR model and then use the top 5% time-points of the test data to test the model. Similarly we perform experiments for bottom 5% values as well. Table 10 shows the comparison for the three brain disorder dataset. The results show that the LR model provides high AUC score by just using 5% of the important time-points. Thus, it proves that a) not all time-points are important for classification of the downstream task and b) our model accurately finds the important time-points. We use an LR model for this experiment to show that the learned top and bottom 5% values are not limited to our DICE model but is generalized such that an independent LR module gives high classification performance using the top 5% data identified by our model and does not learn on the low 5% data. Finally, our experiments also show that not using the temporal attention reduces the model classification performance by upto 10% A.12.

## 4. Discussion

Our experiments revealed a number of interesting properties of DICE and uncovered some interpretable directed connectivity graphs that we feel are of high utility for the neuroimaging field. As supported by results, models with glass-box layer like DICE have a high potential for studying resting-state dynamics of the brain. In the following, we discuss the most pertinent results.

### 4.1. Inter-network and directed connectivity

Results in Sections 3.2.1 and 3.2.2 show that DICE infers DNC that agrees with the essential findings of the FC studies (Arslan et al., 2018; Kawahara et al., 2016; Kazi et al., 2021; Kim and Ye, 2020; Ktena et al., 2017; 2018; Ma et al., 2019; Parisot et al., 2018; Yan et al., 2017) and provides two additional aspects: inter-network connectivity and direction of connectivity. The inter-network connectivity is of great significance as the brain is not made up of isolated networks and many tasks require information passing and neurons firing through multiple networks. Thus making it crucial to find how these networks are connected to each other if connected at all for patients and controls. Capturing the dysconnectivity between networks for patients can lead to knowledge discovery about the functionality of the human brain and the effects of brain disorders on it. Furthermore, finding directionality between networks is also of great significance. We showed in experiments that our model captures the direction of connectivity between networks. The direction of connectivity from VI to other networks, and from CC to SM networks is justifiable. Existing studies (Breukelaar et al., 2017; Cole and Schneider, 2007; Tsai et al., 2019) show that cognitive control is responsible for functions like attention, remembering, and execution. These functions are often required when doing a motor task controlled by sensorimotor, which hints at the direct effect of the CC network on the SM network, captured by DICE. Regarding VI and other networks, we know that VI is mostly a means of input (visuals) to our brain, which is then processed by different parts of the brain. Thus, most of the flow of information is from VI to other networks and few in the opposite direction, which is required to control VI for accomplishing different motor tasks controlled by SM. Therefore, our experiments also show that most incoming connections to VI are through the SM network, thus accurately capturing the flow of information between networks. This flow of information is not captured in simple correlations. We believe these two aspects are crucial to understanding brain working and are currently missed in connectivity estimation methods such as FNC.

Directed connectivity directed influence of an intrinsic brain network on other networks. Estimating the direction of connectivity may simplify targeted interventions that are instrumental in establishing causal relations. Capturing causality between networks further helps to understand complex systems and answer counter-factual questions (Schölkopf et al., 2021), and is left to future work. Our model finds non-negative relations between components/nodes, which we consider dependencies or relevance rather than correlations. However, we understand that the negative correlations in FC and FNC are also helpful and provide descriptive information. We think it might be an easy fix to incorporate negative relations in connectivity matrices estimated by DICE. We discuss this in Section Appendix B.

### 4.2. Interpretability

Section 3.2.3 shows how the DC and DNC estimated by DICE are interpretable in how accurately they capture the difference in connectivity between 1) schizophrenia patients and controls and, 2) male and female groups. In classifying schizophrenia patients from controls, our model learned the most significant differences were in the VI, SM, and DM networks. Controls show robust connectivity of VI and SM with each other and with other networks, which is missing for SZ patients. The finding of dysconnectivity and/or lower connectivity scores for VI and SM networks for SZ patients is not surprising as there exists ample evidence in prior studies of schizophrenia leading to multiple abnormalities related to visual and motor functions such as perception of contrast and motion, detection of visual contours, and control of eye movements to name a few (Butler et al., 2008; Chen et al., 1999; Kéri et al., 2002; Silverstein and Rosen, 2015). These abnormalities certainly affect motor skills which we feel is a reason for the low connectivity for SM and VI networks captured by our model for SZ patients. DICE also captures hyper-connectivity in DMN for SZ patients which is reported by existing studies (Guo et al., 2017).

Whereas in classifying gender in the same dataset, DICE emphasized hyper-connectivity in the DM network and hypo-connectivity for the SM network for females compared to males. The differences captured in the DC and DNC for both tasks are supported by existing studies (Culbreth et al., 2021; Filippi et al., 2013; Kim et al., 2021; Lynall et al., 2010; Mak et al., 2016; Morgan et al., 2020; Ritchie et al., 2018; van den Heuvel et al., 2010; Yu et al., 2011; Zhang et al., 2019; Zhu et al., 2020) that show the role of the DMN in gender classification and VI dysconnectivity for schizophrenic patients. Similarly to existing studies (Ingalhalikar et al., 2014; Zhang et al., 2018b), DICE shows that female subjects have higher connectivity between the contralateral homologue brain networks relative to males.

DL models are commonly viewed as black-box models because of the difficulty of interpretation and not easily explained performance on the tasks they are trained on. These models can show excellent performance on tasks such as classification based on the reasons that are not substantially revealing about the input data nor their dynamics. One reason is shortcut learning (Geirhos et al., 2020): a DL model can classify images with or without airplanes with high accuracy by paying attention exclusively to the background (blue sky). Although predictive, such models cannot help in knowledge discovery. To control for shortcut learning we would like to be able to see why predictions are made. One approach is making DL model interpretable. For that a posthoc method is often used, e.g., saliency maps (Angelov et al., 2021; Lewis et al., 2021; Ras et al., 2021; Simonyan et al., 2014). Such methods explain the input data by finding which part(s) of the input the model is most sensitive to. Saliency maps have shown some good results in computer vision tasks in 2d images. The use of saliency maps in neuroimaging and temporal data has different challenges (Liu et al., 2021) as the output maps are noisy, difficult to interpret and does not provide good boundaries nor the connection between different salient regions. Selection of the method for obtaining saliency maps is also something to consider as some of the methods are architecture based. Hence, using saliency maps to get task-specific brain’s connectivity graph is not feasible using current methods. To overcome the black-box nature of DL models and avoid using a posthoc method, we focused on the interpretability of the model’s results. For this purpose, as brain disorders are commonly associated with disruptions in the connectivity pattern of brain networks, we use only the learned connectivity matrices by our model for the downstream classification or prediction tasks, thus making the model extract the abnormality in connectivity relevant to the ground-truth signal. One way to conceptualize about our approach is to think of the generated DC and DNC as a “glass-box layer” (clear and interpretable) layer as noted in Fig. 1. This approach combines flexibility (the layer is trainable) with interpretability and enables the model to capture differences in the connectivity of the groups in classification task. Regression is also possible with our approach, although we leave it for the future work. Our “glass-box layer” approach enables learning the essential networks and their connection to other networks relevant to the training signal and directly output that without using a posthoc method. As the DC and DNCs estimated by our model are based on learnable functions, the output matrices can have slightly different values when the model is retrained, which is an attribute of DL models. Therefore, all the connectivity matrices shown in the paper are averaged over several randomly-seeded trials.

### 4.3. Task-dependent flexible DNC

We fully utilize the flexibility of our DL model to learn task-dependent (ground-truth signal) directed connectivity structures. We show in Section 3.2.4 that our model estimates DNC structures for the same subjects that are distinct to the ground-truth task of dementia, age, or gender. Hence our model can show the networks and their connectivity crucial for specific downstream tasks. The networks identified by the model through the learned DNC for dementia classification (SM, CB, VI) match the results of prior studies (Albers et al., 2015; Filippi et al., 2017; Grant et al., 2014; Ingalhalikar et al., 2014; Jacobs et al., 2017). Whereas, for gender prediction, the most prominent network identified by the network was DM, which again matches existing literature (Filippi et al., 2013; Kim et al., 2021; Mak et al., 2016; Ritchie et al., 2018). We feel this is a strong validation of the ability of DICE to find disorder-dependent networks and connectivity patterns. We showed in Fig. 8a that our model focused more on SMN than DMN despite having almost two-thirds of female subjects in the test set. This result is significant because the model learned that the SMN connectivity, is more important than DMN for the downstream task of dementia classification and hence enhances the signals for SMN. This eliminates the need to acquire strictly matched subjects with only the difference(s) for which you want to find the relevant networks and connectivity. For example, when trying to find the networks related to schizophrenia using PCC, one needs to find two groups (schizophrenia patients and controls) that do not have any other differences. Extraneous differences would create ambiguity regarding whether the networks identified are related to the disorder (schizophrenia) or some other difference, e.g., gender. Instead of explicitly confronting the confounding factors by regressing them out or taking equivalent measures, DICE performs the “de-confounding” implicitly based on the training labels.

Another notable property of our model is that it finds the relevant networks and the connectivity structures (sub-graphs) without receiving them during training, making DICE a self-supervised graph learning model.

### 4.4. Dynamic DNC and temporal-attention

As hypothesized, and shown in previous studies (Allen et al., 2012; Calhoun et al., 2014; Hutchison et al., 2013; Sakoğlu et al., 2010) results in Section 3.2.5 show that connectivity between brain’s intrinsic network is dynamic, and dynamic connectivity can capture patterns which are missed by static models. Notably, controls and SZ patients spend different amounts of time in each state 10. Controls spend more time than SZ patients in strongly connected states, especially for visual and sensorimotor networks. On the other hand, SZ patients spend time in weakly connected states and do not often spend time in other states. Similar patterns were observed in FNC studies (Damaraju et al., 2014; Rabany et al., 2019; Rashid et al., 2014; Wang et al., 2014; Yang et al., 2022).

Moreover, using all subjects in the FBIRN (Keator et al., 2016), our model finds additional states doubling the state resolution. We explain this temporal resolution increase by instantaneity of directed connectivity estimation in DICE in contrast to using a sliding window. Therefore, estimating connectivity instantaneously makes the model robust and finds patterns that are missed when using a window-based approach. Another explanation and an additional factor is the increased richness of representation via a directed graph - the connectivity matrices of DICE have twice the number of parameters compared to FC and FNC. Our experiment with *k*=*10* states show similar patterns of strongly and weakly connected states but they now vary in the direction of the connectivity. This result shows that both the connectivity strength and direction of connectivity are dynamic (changes over time). As this state is rare (based on time spent), it would be harder for window-based approaches to capture it. It would be interesting to see when and how the direction of connectivity changes and how external factors like performing a task can trigger these changes. This, however, is a topic of the future work.

Finally, we show that not all time-points of the fMRI data are equally important to the downstream prediction task and discriminative connectivity matrices exhibit temporal dynamics. Using temporal attention, our model finds important time-points relevant to the ground-truth signal used in training. This further helps in interpretability as our model finds the time-points where the brain activity shows signals relevant to the task. Potentially, this would also be important in task data where the subject is asked to perform different tasks, and the DICE model can be used to find out which task revealed the symptoms of the underlying disorder. Our experiments show that temporal attention assigns stable and consistent weights to time-points across different randomly-seeded tasks. We also notice that a) just 5% of time-points are sufficient for achieving high classification performance and b) exclusion of temporal attention (assigning the same weight to every time-point) negatively affects classification performance. Consistent temporal attention values across randomly-seeded trials further strengthens the evidence of temporally dynamic discriminative DCs and the value of attention mechanism. As our experiments show, our attention module is indeed reliable per the definitions and potential issues discussed by Jain and Wallace (2019) and Wiegreffe and Pinter (2019). As a learnable method, DICE and other “glass-box layer” models need to be able to consistently across training runs assign temporal attention values and estimate connectivity between nodes, whereas inflexible methods computing correlations such as PCC do not have this property. In a way, flexibility of the learnable model comes with an additional requirement of stability of learned interpretations. Even though our DICE model works well by showing high classification performance and assigning consistent self and temporal attention values on relatively small datasets, as we show, having more subjects for training leads to an even more consistent assignment of temporal weights in our experiments.

## 5. Conclusions

Our work demonstrates importance of learnable interpretable estimators of dynamic, directed, and task-dependent connectivity graphs from fMRI data. DICE learns to estimate interpretable dynamic and directed graphs that represent the directed connectivity among brain networks. The end-to-end training process removes the need for existing external methods such as PCC and K-means, which are interpretable but inflexible and strictly depend on the input data. Implementing DICE with glass-box layer allowed us to bypass the need for a posthoc method for interpreting learned model representations.

Connectivity matrices estimated by DICE show how brain connectivity changes across disorders, genders, and age. The learned connectivity matrices help understand the human brain and its disorders as the actual ground-truth connectivity matrix is not available. Furthermore, we moved from FC and FNC to DC and DNC to learn the direction of connectivity and simultaneously removed the issue of window sizing of input data by making the model instantaneous. The learned connectivity matrices provide knowledge that adheres to existing studies. Utilizing flexibility of DL models in learning data representations, we show that using the same data, distinct connectivity structures can be learned based on the downstream task and the ground-truth signal. This flexibility allows acquiring more information from the data by using different training labels, which would require a much more involved process of data selection and manual filtering out of confounding factors for methods that are fully determined by the data, like PCC. Our model highlights different networks linked with the downstream classification task, e.g., the default mode network for gender prediction. Unlike other interpretable models that may pay for it with a decrease in classification performance (Dhurandhar et al., 2018; Johansson et al., 2011; Luo et al., 2019; Shukla and Tripathi, 2012), DICE beats state of the art methods in multiple classification problems on four neuroimaging datasets.

For classification DICE uses the learned connectivity structures. Together with the temporal weights these structures are reasonably consistent across varying seeds. Notably, DICE’s performance drops without the use of temporal attention. The temporal attention module of the model finds interpretable bio-markers crucial to performing the classification task and shows that only a small fraction of time-points is enough for attaining maximum performance. Notably, not all time points are discriminative, as evident from the sparse distribution of temporal attention weights in Fig. 12 and high predictive power of just the top 5% of the attention weights of Table 10.

As the ground truth for the dynamic graph structure in resting state fMRI is unavailable, we believe there is a need for models with “glass-box layer“ like DICE that can estimate this structure based only on the data and classification labels.

In future work, we would like to omit pre-processing with a dimensionality reduction method—like the used here ICA or region-based parcellation—and train a model end-to-end on the voxel-level data. This, however, may require substantially larger datasets and may not be as useful as the current model for an average sized research dataset. As DICE estimates the direction of connectivity, for future work, we would like to examine how the direction of connectivity changes through time and during tasks for HC and patients.

## Figures and Tables

**Table 1:** Details of the datasets used. We tried different number of test folds in our experiments but that did not have a significant effect on results. Time-points is the number of time-points for each subject in the dataset. Refer to Section Appendix A for more details. In the paper we report the results with test folds that match comparing studies.

Name	Category	Preprocessing	Parcellation	Subjects	0 Class	1 Class	Test Folds	Time-points

FBIRN	Schizophrenia	SPM12	ICA	311	151	160	4,6,**18**	157
OASIS	Dementia	SPM12	ICA	912	651	261	4, **10**	157
ABIDE	Autism	SPM12	ICA	569 (TR=2)	255	314	5, **10**	140
ABIDE	Autism	SPM12	ICA	869	398	471	5, **10**	140
HCP	Gender	SPM12	ICA	833	390	443	5, **15**	980
FBIRN	Schizophrenia	SPM12	Shaefer 200	311	151	160	18	157
HCP	Gender	Glasser	Shaeffer 200	942	411	531	10	1200
ABIDE	Autism	C-PAC	Shaeffer 200	871	403	468	10	83–316

**Table 2:** Classification performance comparison of DICE with other DL methods on region-based (ROIs) data of HCP and FBIRN datasets (Ref to Section 2.1.1). Our DICE model outperforms all other methods in almost every metric. The best two scores are shown as bold and italic respectively. **Note:** As we use all the regions in the atlas we report the mean accuracy for SVM-RBF (Weis et al., 2019). The results for GCN (Arslan et al., 2018) on HCP data are reported by GIN (Kim and Ye, 2020). GIN (Kim and Ye, 2020) and ST-GCN (Gadgil et al., 2021) use test data as validation data for choosing the best performing model. We would also like to point here a newer version of GIN (Kim and Ye, 2020), named STAGIN (Kim et al., 2021) reports AUC and ACC score of 92.96 and 88.20 respectively using 1093 subjects, and 5-fold testing. STAGIN (Kim et al., 2021) reports much lower ACC for GIN and ST-GCN (81.34 and 76.95 respectively) when not using test data as validation data and keeping other parameters (data, preprocessing, parcellation etc.) same. NA: Not Available.

HCP - Gender Classification							FBIRN	
	
	DICE	GIN	SVM-RBF	GCN	ST-GCN	PLS	DICE	BrainGNN

AUC	**0.935**	NA	NA	NA	NA	*0.881*	**0.825**	*0.788*
ACC (%)	**85.8**	*84.6*	68.7	83.98	83.7	79.9	NA	NA
Precision (%)	*85.7*	**86.19**	NA	*84.59*	NA	NA	NA	NA
Recall (%)	**90.2**	86.81	NA	*87.78*	NA	NA	NA	NA
Parcellation	Shaefer 200	Shaefer 400	Shaefer 400 + Fan 39	Shaefer 400	Multi-modal 22	Dosenbach 160	Shaefer 200	AAL 116
Test Folds	10	10	10	10	5	10	18	18
Subjects	942	942	434	942	1091	820	311	311
Study	Our	Kim and Ye (2020)	Weis et al. (2019)	Arslan et al. (2018)	Gadgil et al. (2021)	Zhang et al. (2018a)	Our	Mahmood et al. (2021)

**Table 3:** Comparison of AUC score on ABIDE region-based (ROIs) dataset. Existing methods use Harvard Oxford (HO) parcellation with 111 brain regions, therefore we tested DICE using two atlases. Unlike Parisot et al. (2018), Cao et al. (2021) we use only fMRI data. We also show that DICE model doesn’t depend on the region atlas and gives similar performance using different atlases for region parcellation of the brain.

Method	Parcellation	Input	n_regions	AUC

DICE	Shaefer	fMRI data	200	*0.70*
DICE	HO	fMRI data	111	0.69
GCN (Parisot et al., 2018)	HO	fMRI + phenotypic data	111	**0.75**
DeepGCN (Cao et al., 2021)	HO	fMRI + phenotypic data	111	**0.75**
Metric Learning (Ktena et al., 2018)	HO	fMRI data	111	0.58

**Table 4:** We compare the D/FNCs on the basis of AUC score on FBIRN dataset. We train and test a logistic regression (LR) model using FNCs computed by PCC, and using DNCs estimated by DICE. Performance using estimated DNCs is in reaching distance of ML methods using hand-crafted features (FCs). Appendix A show some experiment details that lead to an even improved classification results.

Method	Input	Mean	Max	Min	Std Dev

LR	PCC FNC	0.883	1	0.72	0.085
LR	Our DNC	0.86	1	0.62	0.096

**Table 5:** Shows stats between male and female DNCs (6 b) estimated using ICA time-courses of OASIS. We see that the estimated DNCs for male and female subjects are highly significantly different. For females DMN is hyper-connected than SMN whereas for male SMN has higher average connectivity score than DMN. This shows that the model accurately captures the group differences among male and female subjects and uses the connectivity difference in DMN and SMN to classify male and female subjects. F - Female, M - Male, All - All networks/complete matrix. Results of classification performance is shown in Table 9. Table 6 shows the *p*-value significance ranges.

Network 1	Network 2	Test Type	*P*-value	Avg. Connectivity 1	Avg. Connectivity 2

		*t*-test	1e-250		
F_All	M_All	manwhitneyu	1e-256	0.353	0.311
		*t*-test	0.15		
F_DM	F_SM	manwhitneyu	0.12	0.536	0.510
		*t*-test	5e-5		
M_DM	M_SM	manwhitneyu	4e-5	0.417	0.575
		*t*-test	6e-4		
F_DM	M_DM	manwhitneyu	4e-4	0.536	0.417
		*t*-test	3e-4		
F_SM	M_SM	manwhitneyu	5e-5	0.510	0.575

**Table 6:** Ranges of *p*-value and the corresponding significance level. ns (no significance).

*P*-value	*p* > 0.10	0.05 < *p* < 0.10	0.01 < *p* < 0.05	0.005 < *p* < 0.01	0.0001 < *p* < 0.005	*p* < 0.0001

Significance	ns	*	**	***	****	*****

**Table 7:** Shows stats between male and female DCs (7 b) estimated using region-based (ROIs) HCP dataset. We clearly see that females have hyper-connectivity in DMN and hypo-connectivity in SMN as compare to males. Female group has higher connectivity scores in DMN compared to SMN and male DMN whereas male group has higher connectivity in SMN compared to DMN and female SMN. This shows that our learned model accurately captures the differences in DMN and SMN connectivity among males and females and uses that for classification. F - Female, M - Male, L - Left, R - Right. Table 6 shows the *p*-value significance ranges.

Network 1	Network 2	Test Type	*P*-value	Avg. Connectivity 1	Avg. Connectivity 2

F_All	M_All	*t*-test	1e-14	0.455	0.533
		manwhitneyu	1e-25		
F_L_DM_temp	F_L_SM	*t*-test	2e-3	0.689	0.632
		manwhitneyu	4e-3		
F_R_DM_temp	F_R_SM	*t*-test	7e-4	0.671	0.593
		manwhitneyu	4e-4		
M_L_DM_temp	M_L_SM	*t*-test	2e-7	0.567	0.622
		manwhitneyu	1e-3		
M_R_DM_temp	M_R_SM	*t*-test	9e-4	0.558	0.611
		manwhitneyu	2e-4		
F_L_DM_temp	M_L_DM_temp	*t*-test	4e-5	0.689	0.567
		manwhitneyu	6e-5		
F_R_DM_temp	M_R_DM_temp	*t*-test	8e-5	0.671	0.558
		manwhitneyu	3e-5		
F_L_DM_pCunPCC	F_L_SM	*t*-test	2e-4	0.718	0.632
		manwhitneyu	1e-3		
F_R_DM_pCunPCC	F_R_SM	*t*-test	1e-5	0.758	0.593
		manwhitneyu	5e-5		
M_L_DM_pCunPCC	M_L_SM	*t*-test	2e-7	0.548	0.622
		manwhitneyu	3e-4		
M_R_DM_pCunPCC	M_R_SM	*t*-test	1e-2	0.547	0.611
		manwhitneyu	1e-2		
F_L_DM_pCunPCC	M_L_DM_pCunPCC	*t*-test	2e-4	0.718	0.548
		manwhitneyu	3e-4		
F_R_DM_pCunPCC	M_R_DM_pCunPCC	*t*-test	3e-4	0.758	0.547
		manwhitneyu	7e-4		
F_L_SM	M_L_SM	*t*-test	1e-1	0.632	0.622
		manwhitneyu	4e-1		
F_R_SM	M_R_SM	*t*-test	1e-2	0.593	0.611
		manwhitneyu	2e-3		

**Table 8:** We compute the statistical difference of the learned connectivity matrices for OASIS ICA when predicting dementia, age and gender. The results show that the learned connectivity matrices are highly statistically different and SMN gets higher connectivity scores than DMN for dementia prediction whereas the opposite is seen for gender prediction.

Network 1	Network 2	Test Type	*P*-value	Avg. Connectivity 1	Avg. Connectivity 2

Dementia_All	Age_All	*t*-test	5e-22	0.323	0.168
		manwhitneyu	1e-38		
Dementia_All	Gender_All	*t*-test	2e-3	0.323	0.311
		manwhitneyu	8e-4		
Age	Gender	*t*-test	2e-301	0.168	0.311
		manwhitneyu	1e-301		
Dementia_DM	Dementia_SM	*t*-test	1e-7	0.478	0.645
		manwhitneyu	8e-8		
Age_DM	Age_SM	*t*-test	6e-1	0.294	0.308
		manwhitneyu	6e-2		
Gender_DM	Gender_SM	*t*-test	4e-1	0.527	0.555
		manwhitneyu	1e-1		
FNC_DM	FNC_SM	*t*-test	3e-2	0.487	0.580
		manwhitneyu	7e-3		
Dementia_DM	Age_DM	*t*-test	9e-6	0.478	0.294
		manwhitneyu	5e-7		
Dementia_DM	Gender_DM	*t*-test	2e-1	0.478	0.527
		manwhitneyu	1e-1		
Age_DM	Gender_DM	*t*-test	3e-7	0.294	0.527
		manwhitneyu	5e-8		
Dementia_SM	Age_SM	*t*-test	8e-34	0.645	0.308
		manwhitneyu	3e-23		
Dementia_SM	Gender_SM	*t*-test	4e-4	0.645	0.555
		manwhitneyu	1e-4		
Age_SM	Gender_SM	*t*-test	1e-18	0.308	0.555
		manwhitneyu	4e-17		

**Table 9:** Dementia, gender classification and age prediction results on OASIS dataset. We compare our results with ML methods using FC computed via PCC. Even with hand-crafted features ML methods perform similarly as our model. We believe the same input because of FC being only data dependent is one of the reasons of ML methods performing lower than DICE for Dementia and age prediction.

Dataset	Model	Task	N_Folds	Input	Metric	Score

OASIS	DICE	Dementia classification	10	ICA	AUC	**0.752**
OASIS	Logistic Regression	Dementia classification	10	FNC	AUC	0.745
OASIS	DICE	Gender classification	10	ICA	AUC	0.906
OASIS	Logistic Regression	Gender classification	10	FNC	AUC	**0.948**
OASIS	DICE	Age prediction	10	ICA	MAE	**6.14**
OASIS	Linear Regression	Age prediction	10	FNC	MAE	7.17
OASIS	Lasso	Age prediction	10	FNC	MAE	6.89

**Table 10:** AUC score comparison on brain datasets with ICA components by using all, top 5% and bottom 5% time-points only. We train and test a logistic regression (LR) model using the time-points identified by DICE and compare the results when using top and bottom 5% time-points. We see that using only top 5% time-points are enough to almost reach the AUC using all time-points.

	Method	FBIRN	OASIS	ABIDE

100%	DICE	0.86	0.752	0.722
Top 5%	LR	0.85	0.743	0.706
Bottom 5%	LR	0.566	0.548	0.532

**Fig. B1.**
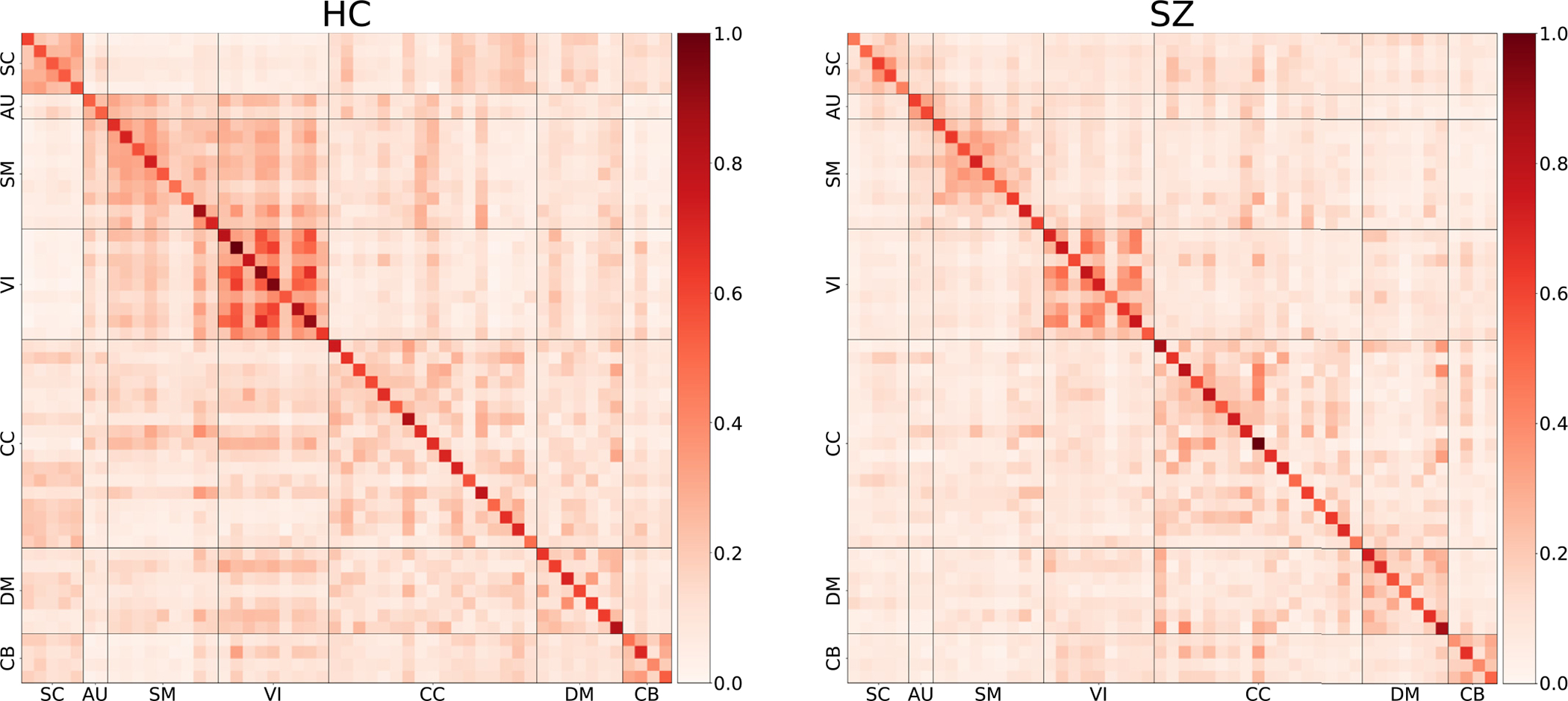
DNC estimated by DICE model using the loss Eq. (B.1). We used the same FBIRN subjects as in Fig. 6a.

**Fig. B2.**
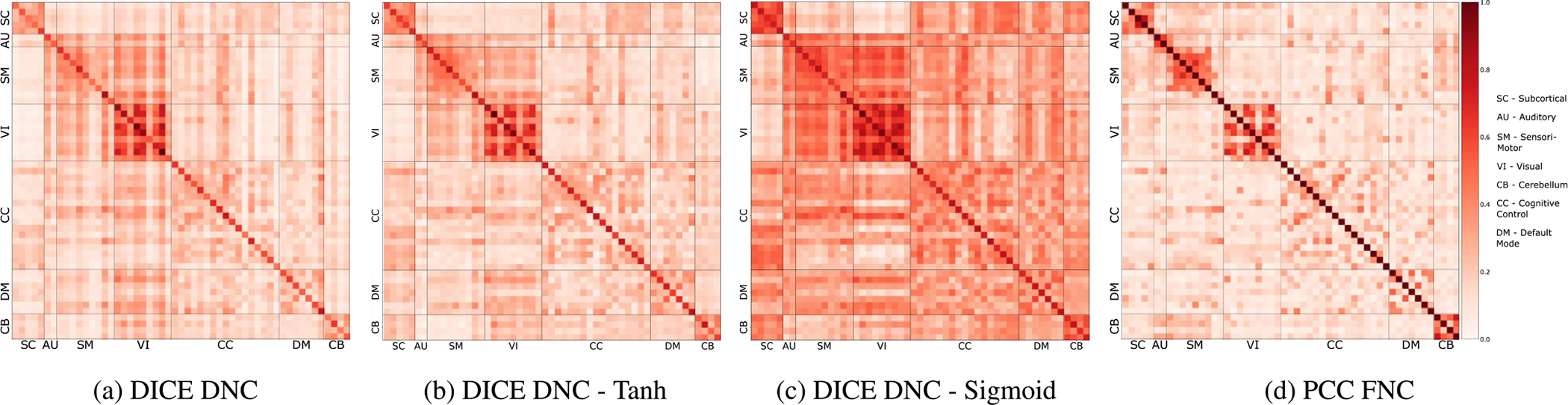
Comparison of the DNCs learned with the additional regularization terms in the loss function against the DNC created using original loss and PCC FNC. As expected, regularization pushes the diagonal closer to 1. Also the difference between values of diagonal and non-diagonal elements is higher in tanh based DNC B.14 b as compared to sigmoid based DNC B.14 c. Similarly to Fig. 4 these matrices are averaged across multiple tries.

**Fig. B3.**
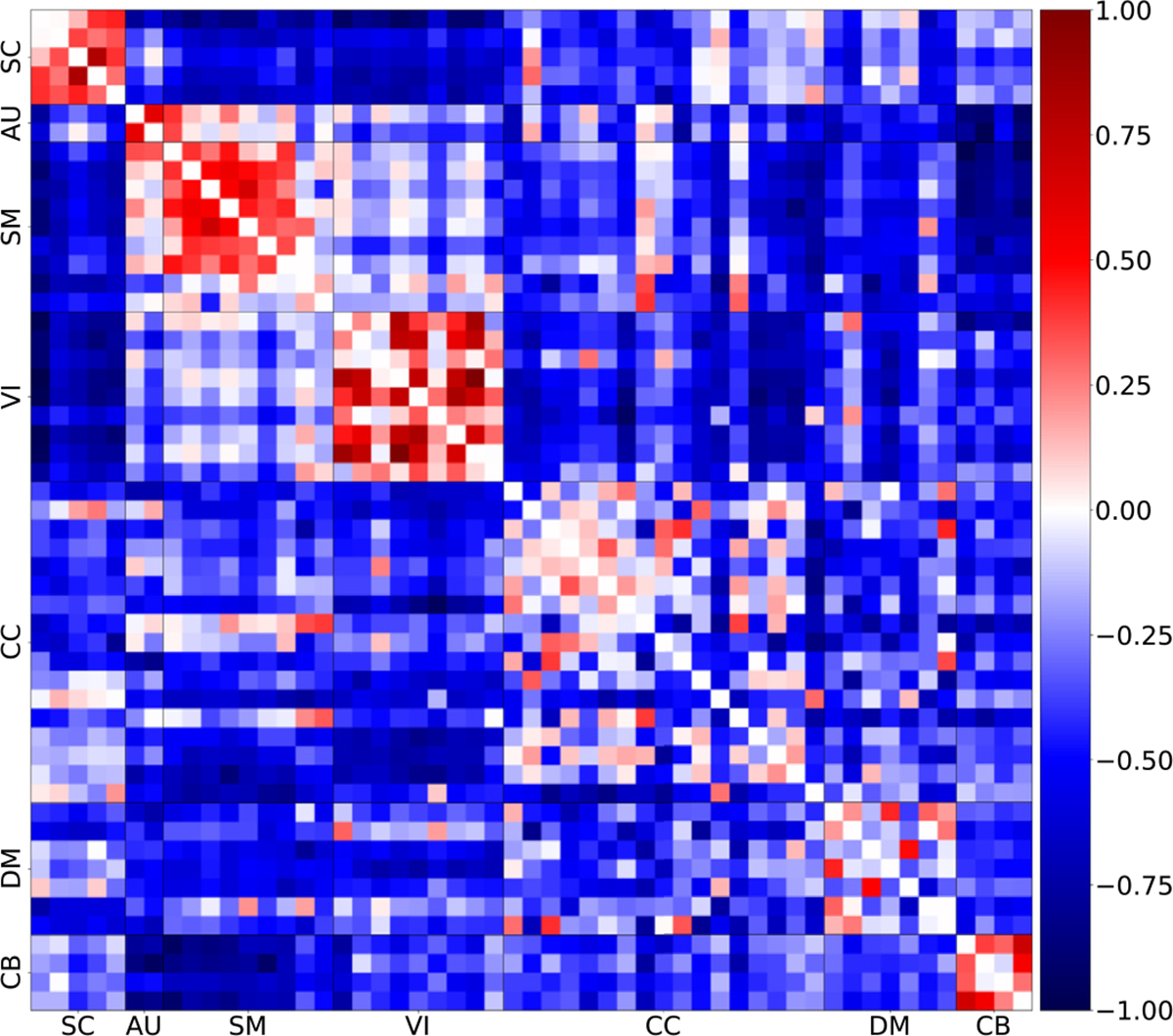
DNC estimated by DICE model by incorporating negative weights in self-attention module. We used the same FBIRN subjects as in Fig. 4a. The diagonal is manually assigned 0 weight.

## Data Availability

This study does not introduce a new dataset and all datasets used in this study are properly referenced. The code for the DICE model is available here https://github.com/UsmanMahmood27/DICE. Machine learning models’ results are computed using python package Polyssifier, available at https://github.com/alvarouc/polyssifier

## Footnotes

1 We use FBIRN phase III.

2
http://fcon_1000.projects.nitrc.org/indi/abide/

3
https://www.oasis-brains.org/

4
https://trendscenter.org/data/

5
https://github.com/alvarouc/polyssifier

## Appendix A. Ablation study

In this section, we show the stability of our DICE model in terms of classification performance by changing different hyper-parameters. We also show that as we did not extensively fine-tune the model for different experiments, it is possible to achieve better classification performance than reported in the paper. In Table A.11 we show the effect of number of test folds on classification performance. Table A.12 shows the effect on performance when changing the size of hidden dimensions. Also, as FBIRN experiments with 18 fold testing created the biggest leakage, the experiment without leakage was necessary for completeness and shows model performs similarly. All other experiments had leakage of 1–2 subjects whose effect should be insignificant. In Table A.13, we show that it is possible to get a bit different classification results than ones reported in the main body by permuting the subjects in different order.

**Table A1:** We show the effect of the different number of test folds on the classification performance of the model using ICA data. We also do an experiment (18, no leakage) where the last fold had all the remaining subjects to prevent any data leakage. We see that the model shows similar performance on different number of test folds with an increase in performance with a greater number of folds.

Dataset	Number of test folds	Mean AUC	Median AUC

FBIRN	4	0.859	0.861
FBIRN	18	0.86	0.861
FBIRN	18, no leakage	0.86	0.861
ABIDE	5	0.705	0.71
ABIDE	10	0.722	0.732
OASIS	5	0.741	0.749
OASIS	10	0.752	0.758

**Table A2:** We show how hidden dimensions of different modules of the model affect classification performance. As we do not fine-tune the hyper-parameters rigorously for each experiment, it is possible to get better results than ones reported in the main body of the paper. Similar results were seen for other datasets as well. We also show how removing the temporal attention reduces the model’s classification performance. None means the final connectivity matrix **W***^f^* was just the average of each **W***_t_*.

Dataset	biLSTM dim.	Self-attention dim.	*γ*_2_	Temporal Attention	Mean AUC	Median AUC

FBIRN	100	48	0.05	GTA	0.86	0.861
FBIRN	100	48	0.05	None	0.733	0.764
FBIRN	100	64	0.05	GTA	0.858	0.861
FBIRN	128	64	0.025	GTA	0.865	0.875
FBIRN	128	64	0.025	None	0.761	0.778
FBIRN	64	32	0.05	GTA	0.849	0.858

**Table A3:** We show how permuting the order of the subjects can lead to a small variation in the classification performance.

Dataset	biLSTM dimension	Self-attention dimension	*γ*_2_	Permutation	Mean AUC	Median AUC

FBIRN	100	48	0.05	Randomly done	0.86	0.861
FBIRN	128	64	0.025	Randomly done	0.865	0.875
FBIRN	100	48	0.05	Default order	0.86	0.889
FBIRN	128	64	0.025	Default order	0.858	0.875

## Appendix B. Added loss term and DNC with negative weights

Connectivity of a node with itself equal to one is the only known and correct bias we can use while estimating connectivity matrix between nodes. Therefore we experimented by adding a new loss term in Eq. (5) and create following two variations.

(B.1)
$$loss\;=\;\text{CrossEntropy}\;(\hat{y},y)+\beta \left(1-\frac{1}{N}\mathrm{tr}\left(\tanh\left(W^{f}\right)\right)\right)+\lambda \|\theta {\|}_{1}$$

(B.2)
$$loss\;=\;\text{CrossEntropy}(\hat{y},y)+\beta \left(1-\frac{1}{N}\mathrm{tr}\left(\mathrm{sigmoid}\left(W^{f}\right)\right)\right)+\lambda \|\theta {\|}_{1}$$

The second term in Eqs. (B.1) and (B.2) is used to encourage the model to produce connectivity matrices with the average value of the main diagonal closer to 1. tr represents the trace of a matrix. *β* is a regularization coefficient and we kept it at 0.75. *β* equal to 1 does push the diagonal closer to 1 but leads to reduction in classification performance. We found in our experiments that the second term results in more stable and easier to visualize matrices across multiple trials. The added term did not significantly affect the classification performance as shown in Table B.14 with tanh and sigmoid activation. Figure B.13 shows the same matrix as Fig. 6a created with the new loss Eq. (B.1).

We also re-create Fig. 4 using the new loss Eqs. (B.1) and (B.2) and show the estimated DNC in Fig. B.14. The added loss terms noticeably increase the values on the diagonal of the connectivity matrices closer to 1. Notably, the difference between diagonal and non-diagonal values is higher in DNC with tanh loss term than sigmoid based DNC. We expect that this is probably because the output value for non-negative input (0) in sigmoid is 0.5 and not 0 as in tanh. Hence, the loss for sigmoid is in the range [0–0.5] and not [0–1]. The choice of the function depends on the application and factors such as the presence of self edges, negative edges, the range of the edge weights etc.

**Table B1:** We compare the classification performance on FBIRN ICA data with the new term added in the loss function. There is not a significant difference in performance, though marginal improvement is seen with sigmoid activation.

Dataset	Added loss term	Mean AUC	Median AUC

FBIRN	None	0.86	0.861
FBIRN	tanh	0.859	0.861
FBIRN	sigmoid	0.862	0.875

As FC and FNC are computed using PCC method to measure the correlations, it has negative correlations as well. These negative correlations are used in different studies and have meaningful interpretations. Therefore, we try to accommodate negative values in the DC and DNC estimated by our model. This can be done easily by making a small tweak in the self-attention part of the model. Equation (2) uses softmax function to get the weights and forces them in the range 0–1. Negative weights can be achieved by replacing the softmax function with tanh. We recreate Fig. 4a by estimating negative weights as well. We see in Fig. B.15 that DICE can capture the negative weights by making a small tweak in the self-attention part but detail experiments are required to check the classification performance, stability, and interpretation if negative weights are incorporated. Also, incorporating negative weights require some hyper-parameter changes as well. We leave this for future work.

## References

[R1] Abraham A, Milham MP, Di Martino A, Craddock RC, Samaras D, Thirion B, Varoquaux G, 2017. Deriving reproducible biomarkers from multi-site resting-state data: an autism-based example. Neuroimage 147, 736–745. doi:10.1016/j.neuroimage.2016.10.045. 27865923

[R2] Aertsen A, Preissl H, 1991. Dynamics of activity and connectivity in physiological neuronal networks. Nonlinear Dyn. Neuronal Netw 281–302.

[R3] Albers MW, Gilmore GC, Kaye J, Murphy C, Wingfield A, Bennett DA, Boxer AL, Buchman AS, Cruickshanks KJ, Devanand DP, Duffy CJ, Gall CM, Gates GA, Granholm AC, Hensch T, Holtzer R, Hyman BT, Lin FR, Mc-Kee AC, Morris JC, Petersen RC, Silbert LC, Struble RG, Trojanowski JQ, Verghese J, Wilson DA, Xu S, Zhang LI, 2015. At the interface of sensory and motor dysfunctions and Alzheimer’s disease. Alzheimers Dement 11 (1), 70–98. [DOI: 10.1016/j.jalz.2014.04.514] 25022540 PMC4287457

[R4] Allen E, Damaraju E, Plis S, Erhardt E, Eichele T, Calhoun V, 2012. Tracking whole-brain connectivity dynamics in the resting state. Cereb. Cortex doi:10.1093/cercor/bhs352. PMC3920766 23146964

[R5] Allen E, Erhardt E, Damaraju E, Gruner W, Segall J, Silva R, Havlicek M, Rachakonda S, Fries J, Kalyanam R, Michael A, Caprihan A, Turner J, Eichele T, Adelsheim S, Bryan A, Bustillo J, Clark V, Feldstein Ewing S, Filbey F, Ford C, Hutchison K, Jung R, Kiehl K, Kodituwakku P, Komesu Y, Mayer A, Pearlson G, Phillips J, Sadek J, Stevens M, Teuscher U, Thoma R, Calhoun V, 2011. A baseline for the multivariate comparison of resting-state networks. Front. Syst. Neurosci. 5, 2. doi:10.3389/fnsys.2011.00002. 21442040 PMC3051178

[R6] Allen EA, Erhardt EB, Damaraju E, Gruner W, Segall JM, Silva RF, Havlicek M, Rachakonda S, Fries J, Kalyanam R, , 2011. A baseline for the multivariate comparison of resting-state networks. Front. Syst. Neurosci. 5, 2. 21442040 10.3389/fnsys.2011.00002 PMC3051178

[R7] Angelov PP, Soares EA, Jiang R, Arnold NI, Atkinson PM, 2021. Explainable artificial intelligence: an analytical review. WIREs Data Min. Knowl. Discovery 11 (5), e1424. doi:10.1002/widm.1424.

[R8] Armstrong CC, Moody TD, Feusner JD, McCracken JT, Chang S, Levitt JG, Piacentini JC, O’Neill J, 2016. Graph-theoretical analysis of resting-state fMRI in pediatric obsessive–compulsive disorder. J. Affect. Disord. 193, 175–184. doi:10.1016/j.jad.2015.12.071. 26773910 PMC5767329

[R9] Arslan S, Ktena SI, Glocker B, Rueckert D, 2018. Graph saliency maps through spectral convolutional networks: application to sex classification with brain connectivity. arXiv:1806.01764.

[R10] Bielza C, Larranaga P, 2014. Bayesian networks in neuroscience: a survey. Front. Comput. Neurosci. 8, 131. doi:10.3389/fncom.2014.00131. 25360109 PMC4199264

[R11] Breukelaar IA, Antees C, Grieve SM, Foster SL, Gomes L, Williams LM, Korgaonkar MS, 2017. Cognitive control network anatomy correlates with neurocognitive behavior: a longitudinal study. Hum. Brain Mapp. 38 (2), 631–643. 27623046 10.1002/hbm.23401 PMC5347905

[R12] Butler P, Silverstein S, Dakin S, 2008. Visual perception and its impairment in schizophrenia. Biol. Psychiatry 64, 40–47. doi:10.1016/j.biopsych.2008.03.023. 18549875 PMC2435292

[R13] Calhoun V, Miller R, Pearlson G, Adalı T, 2014. The chronnectome: time-varying connectivity networks as the next frontier in fMRI data discovery. Neuron 84 (2), 262–274. doi:10.1016/j.neuron.2014.10.015. 25374354 PMC4372723

[R14] Cao M, Yang M, Qin C, Zhu X, Chen Y, Wang J, Liu T, 2021. Using DeepGCN to identify the autism spectrum disorder from multi-site resting-state data. Biomed. Signal Process. Control 70, 103015. doi:10.1016/j.bspc.2021.103015.

[R15] Chen Y, Nakayama K, Levy D, Matthysse S, Holzman P, 1999. Psychophysical isolation of a motion-processing deficit in schizophrenics and their relative and its association with impaired smooth pursuit. Proc. Natl. Acad. Sci. 96. doi:10.1073/pnas.96.8.4724. PMC16399 10200329

[R16] Chiang S, Guindani M, Yeh HJ, Haneef Z, Stern JM, Vannucci M, 2017. Bayesian vector autoregressive model for multi-subject effective connectivity inference using multi-modal neuroimaging data. Hum. Brain Mapp. 38 (3), 1311–1332. 27862625 10.1002/hbm.23456 PMC6827879

[R17] Chickering D, 2002. Optimal structure identification with greedy search. J. Mach. Learn. Res. 3, 507–554. doi:10.1162/153244303321897717.

[R18] Chickering DM, 2002. Learning equivalence classes of bayesian-network structures. J. Mach. Learn. Res. 2, 445–498. doi:10.1162/153244302760200696.

[R19] Cole MW, Schneider W, 2007. The cognitive control network: integrated cortical regions with dissociable functions. Neuroimage 37 (1), 343–360. doi:10.1016/j.neuroimage.2007.03.071. 17553704

[R20] Culbreth A, Wu Q, Chen S, Adhikari B, Gold J, Waltz J, 2021. Temporal-thalamic and cingulo-opercular connectivity in people with schizophrenia. NeuroImage Clinical 29, 102531. doi:10.1016/j.nicl.2020.102531. 33340977 PMC7750447

[R21] Damaraju E, Allen E, Belger A, Ford J, McEwen S, Mathalon D, Mueller B, Pearlson G, Potkin S, Preda A, Turner J, Vaidya J, van Erp T, Calhoun V, 2014. Dynamic functional connectivity analysis reveals transient states of dysconnectivity in schizophrenia. NeuroImage Clinical 5, 298–308. doi:10.1016/j.nicl.2014.07.003. 25161896 PMC4141977

[R22] Deshpande G, Santhanam P, Hu X, 2011. Instantaneous and causal connectivity in resting state brain networks derived from functional MRI data. Neuroimage 54 (2), 1043–1052. 20850549 10.1016/j.neuroimage.2010.09.024 PMC2997120

[R23] Desikan RS, Ségonne F, Fischl B, Quinn BT, Dickerson BC, Blacker D, Buckner RL, Dale AM, Maguire RP, Hyman BT, Albert MS, Killiany RJ, 2006. An automated labeling system for subdividing the human cerebral cortex on MRI scans into gyral based regions of interest. Neuroimage 31 (3), 968–980. doi:10.1016/j.neuroimage.2006.01.021. 16530430

[R24] Dhurandhar A, Shanmugam K, Luss R, Olsen P, 2018. Improving simple models with confidence profiles. 10.48550/ARXIV.1807.07506

[R25] Di Martino A, Yan C-G, Li Q, Denio E, Castellanos FX, Alaerts K, Anderson JS, Assaf M, Bookheimer SY, Dapretto M, , 2014. The autism brain imaging data exchange: towards a large-scale evaluation of the intrinsic brain architecture in autism. Mol. Psychiatry 19 (6), 659. 23774715 10.1038/mp.2013.78 PMC4162310

[R26] Filippi M, Basaia S, Canu E, Imperiale F, Meani A, Caso F, Magnani G, Falautano M, Comi G, Falini A, Agosta F, 2017. Brain network connectivity differs in early-onset neurodegenerative dementia. Neurology 89 (17), 1764–1772. [DOI: 10.1212/WNL.0000000000004577] 28954876 PMC5664301

[R27] Filippi M, Valsasina P, Misci P, Falini A, Comi G, Rocca M, 2013. The organization of intrinsic brain activity differs between genders: a resting-state fMRI study in a large cohort of young healthy subjects. Hum. Brain Mapp 34. doi:10.1002/hbm.21514. PMC6870508 22359372

[R28] Freedman D, Pisani R, Purves R, 2007. Statistics (International Student Edition), 4th ed. WW Norton & Company, New York.

[R29] Friston K, 2011. Functional and effective connectivity: a review. Brain Connect. 1, 13–36. doi:10.1089/brain.2011.0008. 22432952

[R30] Fu Z, Caprihan A, Chen J, Du Y, Adair JC, Sui J, Rosenberg GA, Calhoun VD, 2019. Altered static and dynamic functional network connectivity in Alzheimer’s disease and subcortical ischemic vascular disease: shared and specific brain connectivity abnormalities. Hum. Brain Mapp.. 10.1002/hbm.24591 PMC6865624 30950567

[R31] Fu Z, Iraji A, Turner JA, Sui J, Miller R, Pearlson GD, Calhoun VD, 2021. Dynamic state with covarying brain activity-connectivity: on the pathophysiology of schizophrenia. Neuroimage 224, 117385. doi:10.1016/j.neuroimage.2020.117385. 32950691 PMC7781150

[R32] Fu Z, Sui J, Turner J, Du Y, Assaf M, Pearlson G, Calhoun V, 2020. Dynamic functional network reconfiguration underlying the pathophysiology of schizophrenia and autism spectrum disorder. Hum. Brain Mapp. 42. doi:10.1002/hbm.25205. PMC7721229 32965740

[R33] Fu Z, Tu Y, Di X, Du Y, Pearlson G, Turner J, Biswal B, Zhang Z, Calhoun V, 2017. Characterizing dynamic amplitude of low-frequency fluctuation and its relationship with dynamic functional connectivity: an application to schizophrenia. Neuroimage 180. doi:10.1016/j.neuroimage.2017.09.035. PMC5860934 28939432

[R34] Fu Z, Tu Y, Di X, Du Y, Sui J, Biswal B, Zhang Z, Lacy N, Calhoun V, 2018. Transient increased thalamic-sensory connectivity and decreased whole-brain dynamism in autism. Neuroimage 190. doi:10.1016/j.neuroimage.2018.06.003. PMC6281849 29883735

[R35] Gadgil S, Zhao Q, Pfefferbaum A, Sullivan EV, Adeli E, Pohl KM, 2021. Spatio-temporal graph convolution for resting-state fMRI analysis. arXiv:2003.10613. 10.1007/978-3-030-59728-3_52 PMC7700758 33257918

[R36] Geirhos R, Jacobsen J-H, Michaelis C, Zemel R, Brendel W, Bethge M, Wichmann FA, 2020. Shortcut learning in deep neural networks. Nat. Mach. Intell. 2 (11), 665–673. doi:10.1038/s42256-020-00257-z.

[R37] Glasser M, Sotiropoulos S, Wilson J, Coalson T, Fischl B, Andersson J, Xu J, Jbabdi S, Webster M, Polimeni J, DC V, Jenkinson M, 2013. The minimal preprocessing pipelines for the human connectome project. Neuroimage 80, 105. doi:10.1016/j.neuroimage.2013.04.127. 23668970 PMC3720813

[R38] Goebel R, Roebroeck A, Kim D-S, Formisano E, 2003. Investigating directed cortical interactions in time-resolved fMRI data using vector autoregressive modeling and Granger causality mapping. Magn. Reson. Imaging 21 (10), 1251–1261. 14725933 10.1016/j.mri.2003.08.026

[R39] Gorrostieta C, Fiecas M, Ombao H, Burke E, Cramer S, 2013. Hierarchical vector auto-regressive models and their applications to multi-subject effective connectivity. Front. Comput. Neurosci. 7, 159. 24282401 10.3389/fncom.2013.00159 PMC3825259

[R40] Grant A, Dennis NA, Li P, 2014. Cognitive control, cognitive reserve, and memory in the aging bilingual brain. Front. Psychol. 5, 1401. [DOI: 10.3389/fpsyg.2014.01401] 25520695 PMC4253532

[R41] Greicius MD, Krasnow B, Reiss AL, Menon V, 2003. Functional connectivity in the resting brain: a network analysis of the default mode hypothesis. Proc. Natl. Acad. Sci. 100 (1), 253–258. 12506194 10.1073/pnas.0135058100 PMC140943

[R42] Griffanti L, Khorshidi GS, Beckmann CF, Auerbach EJ, Douaud G, Sexton CE, Zsoldos E, Ebmeier KP, Filippini N, Mackay CE, Moeller S, Xu J, Yacoub E, Baselli G, Uğurbil K, Miller KL, Smith SM, 2014. ICA-based artefact removal and accelerated fMRI acquisition for improved resting state network imaging. Neuroimage 95, 232–247. 24657355 10.1016/j.neuroimage.2014.03.034 PMC4154346

[R43] Guo W, Liu F, Chen J, Wu R, Li L, Zhang Z, Chen H, Zhao J, 2017. Hyperactivity of the default-mode network in first-episode, drug-naive schizophrenia at rest revealed by family-based case–control and traditional case–control designs. Medicine 96, e6223. doi:10.1097/MD.0000000000006223. 28353559 PMC5380243

[R44] Haan W, Flier W, Koene T, Smits L, Scheltens P, Stam C, 2011. Disrupted modular brain dynamics reflect cognitive dysfunction in Alzheimer’s disease. Neuroimage 59, 3085–3093. doi:10.1016/j.neuroimage.2011.11.055. 22154957

[R45] Hutchison R, Womelsdorf T, Gati S, Everling S, Menon R, 2013. Resting-state networks show dynamic functional connectivity in awake humans and anesthetized macaques. Hum. Brain Mapp. 34. doi:10.1002/hbm.22058. PMC6870538 22438275

[R46] Ingalhalikar M, Smith A, Parker D, Satterthwaite TD, Elliott MA, Ruparel K, Hakonarson H, Gur RE, Gur RC, Verma R, 2014. Sex differences in the structural connectome of the human brain. Proc. Natl. Acad. Sci. 111 (2), 823–828. doi:10.1073/pnas.1316909110. 24297904 PMC3896179

[R47] Jacobs HIL, Hopkins DA, Mayrhofer HC, Bruner E, van Leeuwen FW, Raaijmakers W, Schmahmann JD, 2017. The cerebellum in Alzheimer’s disease: evaluating its role in cognitive decline. Brain 141 (1), 37–47. doi:10.1093/brain/awx194. 29053771

[R48] Jafri MJ, Pearlson GD, Stevens M, Calhoun VD, 2008. A method for functional network connectivity among spatially independent resting-state components in schizophrenia. Neuroimage 39 (4), 1666–1681. 18082428 10.1016/j.neuroimage.2007.11.001 PMC3164840

[R49] Jain S, Wallace BC, 2019. Attention is not explanation. arXiv:1902.10186.

[R50] Jenkinson M, Beckmann CF, Behrens TE, Woolrich MW, Smith SM, 2012. Fsl. Neuroimage 62 (2), 782–790. doi:10.1016/j.neuroimage.2011.09.015. 20 YEARS OF fMRI 21979382

[R51] Johansson U, Sönströd C, Norinder U, Boström H, 2011. Trade-off between accuracy and interpretability for predictive in silico modeling. Future Med. Chem. 3 (6), 647–663. doi:10.4155/fmc.11.23. 21554073

[R52] Kawahara J, Brown C, Miller S, Booth B, Chau V, Grunau R, Zwicker J, Hamarneh G, 2016. BrainNetCNN: convolutional neural networks for brain networks; towards predicting neurodevelopment. Neuroimage 146. doi:10.1016/j.neuroimage.2016.09.046. 27693612

[R53] Kazi A, Farghadani S, Navab N, 2021. IA-GCN: interpretable attention based graph convolutional network for disease prediction. arXiv:2103.15587. 10.1007/978-3-031-45673-2_38 PMC10583839 37854585

[R54] Keator DB, van Erp TG, Turner JA, Glover GH, Mueller BA, Liu TT, Voyvodic JT, Rasmussen J, Calhoun VD, Lee HJ, , 2016. The function biomedical informatics research network data repository. Neuroimage 124, 1074–1079. 26364863 10.1016/j.neuroimage.2015.09.003 PMC4651841

[R55] Kim B-H, Ye JC, 2020. Understanding graph isomorphism network for rs-fMRI functional connectivity analysis. Front. Neurosci. 14, 630. doi:10.3389/fnins.2020.00630. 32714130 PMC7344313

[R56] Kim B-H, Ye JC, Kim J-J, 2021. Learning dynamic graph representation of brain connectome with spatio-temporal attention. arXiv:2105.13495.

[R57] Kipf T, Fetaya E, Wang K-C, Welling M, Zemel R, 2018. Neural relational inference for interacting systems. arXiv:1802.04687.

[R58] Ktena SI, Parisot S, Ferrante E, Rajchl M, Lee M, Glocker B, Rueckert D, 2017. Distance metric learning using graph convolutional networks: Application to functional brain networks. arXiv:1703.02161.

[R59] Ktena SI, Parisot S, Ferrante E, Rajchl M, Lee M, Glocker B, Rueckert D, 2018. Metric learning with spectral graph convolutions on brain connectivity networks. Neuroimage 169, 431–442. doi:10.1016/j.neuroimage.2017.12.052. 29278772

[R60] Kéri S, Antal A, Szekeres G, Benedek G, Janka Z, 2002. Spatio-temporal visual processing in schizophrenia. J. Neuropsychiatry Clin. Neurosci. 14 (2), 190–196. doi:10.1176/jnp.14.2.190. 11983794

[R61] Lee MH, Smyser CD, Shimony JS, 2013. Resting-state fMRI: a review of methods and clinical applications. Am. J. Neuroradiol. 34 (10), 1866–1872. 22936095 10.3174/ajnr.A3263 PMC4035703

[R62] Lewis N, Miller R, Gazula H, Rahman MM, Iraji A, Calhoun VD, Plis S, 2021. Can recurrent models know more than we do? In: 2021 IEEE 9th International Conference on Healthcare Informatics (ICHI), pp. 243–247. doi:10.1109/ICHI52183.2021.00046.

[R63] Liu Z, Adeli E, Pohl KM, Zhao Q, 2021. Going beyond saliency maps: training deep models to interpret deep models. In: International Conference on Information Processing in Medical Imaging. Springer, pp. 71–82. 10.1007/978-3-030-78191-0_6 PMC8451265 34548772

[R64] Luo Y, Tseng H-H, Cui S, Wei L, Ten Haken RK, El Naqa I, 2019. Balancing accuracy and interpretability of machine learning approaches for radiation treatment outcomes modeling. BJR|Open 1 (1), 20190021. doi:10.1259/bjro.20190021. 33178948 PMC7592485

[R65] Lynall M-E, Bassett DS, Kerwin R, McKenna PJ, Kitzbichler M, Muller U, Bullmore E, 2010. Functional connectivity and brain networks in schizophrenia. J. Neurosci. 30 (28), 9477–9487. doi:10.1523/JNEUROSCI.0333-10.2010. 20631176 PMC2914251

[R66] Ma G, Ahmed NK, Willke T, Sengupta D, Cole MW, Turk-Browne NB, Yu PS, 2019. Similarity learning with higher-order graph convolutions for brain network analysis. arXiv:1811.02662.

[R67] Mahmood U, Fu Z, Calhoun VD, Plis S, 2021. A deep learning model for data-driven discovery of functional connectivity. Algorithms 14 (3), 75. doi:10.3390/a14030075.

[R68] Mahmood U, Rahman MM, Fedorov A, Fu Z, Calhoun VD, Plis SM, 2019. Learnt dynamics generalizes across tasks, datasets, and populations. arXiv:1912.03130.

[R69] Mahmood U, Rahman MM, Fedorov A, Lewis N, Fu Z, Calhoun VD, Plis SM, 2020. Whole MILC: generalizing learned dynamics across tasks, datasets, and populations. Lect. Notes Comput. Sci 407–417. doi:10.1007/978-3-030-59728-3_40.

[R70] Mak L, Minuzzi L, MacQueen G, Hall G, Kennedy S, Milev R, 2016. The default mode network in healthy individuals: asystematic review and meta-analysis. Brain Connect. 7. doi:10.1089/brain.2016.0438. 27917679

[R71] Marutho D, Hendra Handaka S, Wijaya E, Muljono, 2018. The determination of cluster number at k-mean using elbow method and purity evaluation on headline news. In: 2018 International Seminar on Application for Technology of Information and Communication, pp. 533–538. doi:10.1109/ISEMANTIC.2018.8549751.

[R72] Miller R, Calhoun V, 2020. Hybrid dictionary learning-ICA approaches built on novel instantaneous dynamic connectivity metric provide new multiscale insights into dynamic brain connectivity. In: Isgum I, Landman B (Eds.), Medical Imaging 2020. SPIE doi:10.1117/12.2549368. Publisher Copyright: © 2020 SPIE. All rights reserved. Copyright: Copyright 2020 Elsevier B.V., All rights reserved.; Medical Imaging 2020: Image Processing; Conference date: 17-02-2020 Through 20-02-2020

[R73] Miller RL, Calhoun VD, 2020. Transient spectral peak analysis reveals distinct temporal activation profiles for different functional brain networks. In: 2020 IEEE Southwest Symposium on Image Analysis and Interpretation (SSIAI), pp. 108–111. doi:10.1109/SSIAI49293.2020.9094609.

[R74] Million E, 2007. The hadamard product.

[R75] Mitra A, Snyder AZ, Hacker CD, Raichle ME, 2014. Lag structure in resting-state fMRI. J. Neurophysiol. 111 (11), 2374–2391. doi:10.1152/jn.00804.2013. 24598530 PMC4097876

[R76] Morgan S, Young J, Patel A, Whitaker K, Scarpazza C, Amelsvoort T, Marcelis M, Os J, Donohoe G, Mothersill D, Corvin A, Arango C, Mechelli A, Heuvel M, Kahn R, McGuire P, Brammer M, Bullmore E, 2020. Functional MRI connectivity accurately distinguishes cases with psychotic disorders from healthy controls, based on cortical features associated with brain network development. Biol. Psychiatry Cogn. Neurosci.Neuroimaging doi:10.1016/j.bpsc.2020.05.013. 32800754

[R77] Parisot S, Ktena SI, Ferrante E, Lee M, Guerrero R, Glocker B, Rueckert D, 2018. Disease prediction using graph convolutional networks: application to autism spectrum disorder and alzheimer’s disease. Med. Image Anal. 48, 117–130. doi:10.1016/j.media.2018.06.001. 29890408

[R78] Pearl J, 2000. Causality, by Judea Pearl, −1. Cambridge University Press, Cambridge, UK, p. 400. ISBN 0521773628

[R79] Rabany L, Brocke S, Calhoun V, Pittman B, Corbera S, Wexler B, Bell M, Pelphrey K, Pearlson G, Assaf M, 2019. Dynamic functional connectivity in schizophrenia and autism spectrum disorder: convergence, divergence and classification. NeuroImage Clinical 24. doi:10.1016/j.nicl.2019.101966. Funding Information: This work has been supported by the National Institutes of Health (NIMH; R01 MH095888; PI: M. Assaf), and the National Alliance for Research in Schizophrenia and Affective Disorders (NARSAD; Young Investigator Award 17525; PI: C. Corbera). Publisher Copyright: © 2019 PMC6700449 31401405

[R80] Ras G, Xie N, van Gerven M, Doran D, 2021. Explainable deep learning: a field guide for the uninitiated. arXiv:2004.14545.

[R81] Rashid B, Damaraju E, Pearlson G, Calhoun V, 2014. Dynamic connectivity states estimated from resting fMRI identify differences among schizophrenia, bipolar disorder, and healthy control subjects. Front. Hum. Neurosci. 8, 897. doi:10.3389/fnhum.2014.00897. 25426048 PMC4224100

[R82] Ritchie SJ, Cox SR, Shen X, Lombardo MV, Reus LM, Alloza C, Harris MA, Alderson HL, Hunter S, Neilson E, Liewald DCM, Auyeung B, Whalley HC, Lawrie SM, Gale CR, Bastin ME, McIntosh AM, Deary IJ, 2018. Sex differences in the adult human brain: evidence from 5216 UK biobank participants. Cereb. Cortex 28 (8), 2959–2975. doi:10.1093/cercor/bhy109. 29771288 PMC6041980

[R83] Rogers BP, Morgan VL, Newton AT, Gore JC, 2007. Assessing functional connectivity in the human brain by fMRI. Magn. Reson. Imaging 25 (10), 1347–1357. 17499467 10.1016/j.mri.2007.03.007 PMC2169499

[R84] Rubin EH, Storandt M, Miller JP, Kinscherf DA, Grant EA, Morris JC, Berg L, 1998. A prospective study of cognitive function and onset of dementia in cognitively healthy elders. Arch. Neurol. 55 (3), 395–401. 9520014 10.1001/archneur.55.3.395

[R85] Sakoğlu Ü, Pearlson G, Kiehl K, Wang Y, Michael A, Calhoun V, 2010. A method for evaluating dynamic functional network connectivity and task-modulation: application to schizophrenia. Magn. Reson. Mater. Phys. Biol. Med. 23 (5–6), 351–366. doi:10.1007/s10334-010-0197-8. PMC2891285 20162320

[R86] Salehi M, Greene AS, Karbasi A, Shen X, Scheinost D, Constable RT, 2020. There is no single functional atlas even for a single individual: functional parcel definitions change with task. Neuroimage 208, 116366. doi:10.1016/j.neuroimage.2019.116366. 31740342

[R87] Salimi-Khorshidi G, Douaud G, Beckmann C, Glasser M, Griffanti L, Smith S, 2014. Automatic denoising of functional MRI data: combining independent component analysis and hierarchical fusion of classifiers. Neuroimage 90. doi:10.1016/j.neuroimage.2013.11.046. PMC4019210 24389422

[R88] Schaefer A, Kong R, Gordon EM, Laumann TO, Zuo X-N, Holmes AJ, Eickhoff SB, Yeo BTT, 2017. Local-global parcellation of the human cerebral cortex from intrinsic functional connectivity MRI. Cereb. Cortex 28 (9), 3095–3114. doi:10.1093/cercor/bhx179. PMC6095216 28981612

[R89] Schreiber T, 2000. Measuring information transfer. Phys. Rev. Lett. 85, 461–464. doi:10.1103/PhysRevLett.85.461. 10991308

[R90] Schuster M, Paliwal K, 1997. Bidirectional recurrent neural networks. IEEE Trans. Signal Process. 45 (11), 2673–2681. doi:10.1109/78.650093.

[R91] Schölkopf B, Locatello F, Bauer S, Ke NR, Kalchbrenner N, Goyal A, Bengio Y, 2021. Towards causal representation learning. arXiv:2102.11107.

[R92] Seth A, Barrett A, Barnett L, 2015. Granger causality analysis in neuroscience and neuroimaging. J. Neurosci. 35, 3293–3297. doi:10.1523/JNEUROSCI.4399-14.2015. 25716830 PMC4339347

[R93] Shukla P, Tripathi S, 2012. A review on the interpretability-accuracy trade-off in evolutionary multi-objective fuzzy systems (EMOFS). Information 3, 256–277. doi:10.3390/info3030256.

[R94] Silverstein SM, Rosen R, 2015. Schizophrenia and the eye. Schizophr. Res. Cognit. 2 (2), 46–55. doi:10.1016/j.scog.2015.03.004. Visual Functioning and Schizophrenia 26345525 PMC4559409

[R95] Simonyan K, Vedaldi A, Zisserman A, 2014. Deep inside convolutional networks: visualising image classification models and saliency maps. arXiv:1312.6034.

[R96] Spirtes P, Glymour C, 1991. An algorithm for fast recovery of sparse causal graphs. Soc. Sci. Comput. Rev. 9, 62–72. doi:10.1177/089443939100900106.

[R97] Spirtes P, Glymour C, Scheines R, 1993. Causation, prediction, and search. Vol. 81. 10.1007/978-1-4612-2748-9

[R98] Supekar K, Cai W, Krishnadas R, Palaniyappan L, Menon V, 2019. Dysregulated brain dynamics in a triple-network saliency model of schizophrenia and its relation to psychosis. Biol. Psychiatry 85 (1), 60–69. doi:10.1016/j.biopsych.2018.07.020. Immune Mechanisms and Psychosis 30177256

[R99] Tsai C-F, Tu P-C, Wang Y, jen Chu C, Huang Y, Lin H-C, Hou M-C, Lee F-Y, Liu P-Y, Lu C-L, 2019. Altered cognitive control network is related to psychometric and biochemical profiles in covert hepatic encephalopathy. Sci. Rep. 9. 10.1038/s41598-019-42957-6 PMC6488566 31036843

[R100] Ursino M, Ricci G, Magosso E, 2020. Transfer entropy as a measure of brain connectivity: a critical analysis with the help of neural mass models. Front. Comput. Neurosci 14. 10.3389/fncom.2020.00045 PMC7292208 32581756

[R101] van den Heuvel MP, Mandl RCW, Stam CJ, Kahn RS, Hulshoff Pol HE, 2010. Aberrant frontal and temporal complex network structure in schizophrenia: a graph theoretical analysis. J. Neurosci. 30 (47), 15915–15926. doi:10.1523/JNEUROSCI.2874-10.2010. 21106830 PMC6633761

[R102] van den Heuvel MP, Pol HEH, 2010. Exploring the brain network: a review on resting-state fMRI functional connectivity. Eur. Neuropsychopharmacol. 20 (8), 519–534. 20471808 10.1016/j.euroneuro.2010.03.008

[R103] van den Heuvel MP, Pol HEH, 2010. Exploring the brain network: a review on resting-state fMRI functional connectivity. Eur. Neuropsychopharmacol. 20, 519–534. doi:10.1016/j.euroneuro.2010.03.008. 20471808

[R104] Van Essen DC, Smith SM, Barch DM, Behrens TE, Yacoub E, Ugurbil K, Consortium W-MH, , 2013. The WU-Minn human connectome project: an overview. Neuroimage 80, 62–79. 23684880 10.1016/j.neuroimage.2013.05.041 PMC3724347

[R105] Vaswani A, Shazeer N, Parmar N, Uszkoreit J, Jones L, Gomez AN, Kaiser u., Polosukhin I, 2017. Attention is all you need. In: Proceedings of the 31st International Conference on Neural Information Processing Systems. Curran Associates Inc., Red Hook, NY, USA, pp. 6000–6010.

[R106] Vicente R, Wibral M, Lindner M, Pipa G, 2011. Transfer entropy–a model-free measure of effective connectivity for the neurosciences. J Comput Neurosci 30 (1), 45–67. 20706781 10.1007/s10827-010-0262-3 PMC3040354

[R107] Wang X, Xia M, Lai Y, Dai Z, Cao Q, Cheng Z, Han X, Yang L, Yuan Y, Zhang Y, Li K, Ma H, Shi C, Hong N, Szeszko P, Yu X, He Y, 2014. Disrupted resting-state functional connectivity in minimally treated chronic schizophrenia. Schizophr. Res. 156 (2), 150–156. doi:10.1016/j.schres.2014.03.033. 24794395

[R108] Weis S, Patil KR, Hoffstaedter F, Nostro A, Yeo BTT, Eickhoff SB, 2019. Sex classification by resting state brain connectivity. Cereb. Cortex 30 (2), 824–835. doi:10.1093/cercor/bhz129. PMC7444737 31251328

[R109] Wiegreffe S, Pinter Y, 2019. Attention is not not explanation. arXiv:1908.04626.

[R110] Xu Da, Ruan C, Korpeoglu E, Kumar S, Achan K, 2020. Inductive representation learning on temporal graphs. In: International Conference on Learning Representations. https://openreview.net/forum?id=rJeW1yHYwH

[R111] Yaesoubi M, Adalı T, Calhoun VD, 2018. A window-less approach for capturing time-varying connectivity in fMRI data reveals the presence of states with variable rates of change. Hum. Brain Mapp. 39 (4), 1626–1636. doi:10.1002/hbm.23939. 29315982 PMC5847478

[R112] Yan W, Plis S, Calhoun VD, Liu S, Jiang R, Jiang T-Z, Sui J, 2017. Discriminating schizophrenia from normal controls using resting state functional network connectivity: a deep neural network and layer-wise relevance propagation method. In: 2017 IEEE 27th International Workshop on Machine Learning for Signal Processing (MLSP). IEEE, pp. 1–6. doi:10.1109/MLSP.2017.8168179.

[R113] Yang W, Xu X, Wang C, Cheng Y, Li Y, Xu S, Li J, 2022. Alterations of dynamic functional connectivity between visual and executive-control networks in schizophrenia. Brain Imaging Behav. doi:10.1007/s11682-021-00592-8. 34997915

[R114] Yao D, Sui J, Yang E, Yap P-T, Shen D, Liu M, 2020. Temporal-adaptive graph convolutional network for automated identification of major depressive disorder using resting-state fMRI. In: Liu M, Yan P, Lian C, Cao X (Eds.), Machine Learning in Medical Imaging. Springer International Publishing, Cham, pp. 1–10. 10.1007/978-3-030-59861-7_1 PMC9645786 36383497

[R115] Yu Q, Sui J, Rachakonda S, He H, Pearlson G, Calhoun V, 2011. Altered small-world brain networks in temporal lobe in patients with schizophrenia performing an auditory oddball task. Front. Syst. Neurosci. 5, 7. doi:10.3389/fnsys.2011.00007. 21369355 PMC3037777

[R116] Zhang C, Dougherty C, Baum S, White T, Michael A, 2018. Functional connectivity predicts gender: evidence for gender differences in resting brain connectivity. Hum. Brain Mapp 39. doi:10.1002/hbm.23950. PMC6866578 29322586

[R117] Zhang J, Dong X, Wang L, Zhao L, Weng Z, Zhang T, Sui J, Go R, Huang Q, Wu J, Yan T, 2018. Gender differences in global functional connectivity during facial emotion processing: a visual MMN study. Front. Behav. Neurosci 12. doi:10.3389/fnbeh.2018.00220. PMC6167960 30319370

[R118] Zhang Y, Dai Z, Chen Y, Sim K, Sun Y, Yu R, 2019. Altered intra- and interhemispheric functional dysconnectivity in schizophrenia. Brain Imaging Behav. 13. doi:10.1007/s11682-018-9935-8. 30094555

[R119] Zhu J, Qian Y, Zhang B, Li X, Bai Y, Li X, Yu Y, 2020. Abnormal synchronization of functional and structural networks in schizophrenia. Brain Imaging Behav. 14. doi:10.1007/s11682-019-00175-8. 31376115

